# Zwitterionically modified alginates mitigate cellular overgrowth for cell encapsulation

**DOI:** 10.1038/s41467-019-13238-7

**Published:** 2019-11-20

**Authors:** Qingsheng Liu, Alan Chiu, Long-Hai Wang, Duo An, Monica Zhong, Alexandra M. Smink, Bart J. de Haan, Paul de Vos, Kevin Keane, Andreas Vegge, Esther Y. Chen, Wei Song, Wendy F. Liu, James Flanders, Claude Rescan, Lars Groth Grunnet, Xi Wang, Minglin Ma

**Affiliations:** 1 Department of Biological and Environmental Engineering, Cornell University, Ithaca, NY 14853 USA; 2 Department of Pathology and Medical Biology, University of Groningen and University Medical Center Groningen, Groningen, Netherlands; 3 Stem Cell Biology, Novo Nordisk A/S, 2760 Måløv, Denmark; 4 Diabetes Research, Novo Nordisk A/S, 2760 Måløv, Denmark; 5 Department of Biomedical Engineering, University of California Irvine, Irvine, CA 92697 USA; 6 Department of Clinical Sciences, Cornell University, Ithaca, NY 14853 USA; 7 Stem Cell Pharmacology, Novo Nordisk A/S, 2760 Måløv, Denmark

**Keywords:** Type 1 diabetes, Implants, Biomedical engineering, Biomaterials

## Abstract

Foreign body reaction (FBR) to implanted biomaterials and medical devices is common and can compromise the function of implants or cause complications. For example, in cell encapsulation, cellular overgrowth (CO) and fibrosis around the cellular constructs can reduce the mass transfer of oxygen, nutrients and metabolic wastes, undermining cell function and leading to transplant failure. Therefore, materials that mitigate FBR or CO will have broad applications in biomedicine. Here we report a group of zwitterionic, sulfobetaine (SB) and carboxybetaine (CB) modifications of alginates that reproducibly mitigate the CO of implanted alginate microcapsules in mice, dogs and pigs. Using the modified alginates (SB-alginates), we also demonstrate improved outcome of islet encapsulation in a chemically-induced diabetic mouse model. These zwitterion-modified alginates may contribute to the development of cell encapsulation therapies for type 1 diabetes and other hormone-deficient diseases.

## Abstract (web summary)

Cellular overgrowth and fibrosis in the foreign body response can compromise the function of transplanted cells. Here, the authors report on the zwitterionically modified alginates for the encapsulation of cells to reduce cellular overgrowth and demonstrate application in mice, dogs and pigs.

## Introduction

Type 1 diabetes (T1D) affects millions of people worldwide, despite that many advanced therapeutic treatments have been developed^1–8^. To date, daily injection or infusion of exogenous insulin is still the leading treatment option to provide blood glucose (BG) control for people with T1D^9^. However, insulin therapies are tedious, often associated with patient compliance and cannot totally prevent diabetic side effects^10^. Pancreatic islet transplantation has worked for some patients^11^, but it is limited to only a small fraction of patients because of a shortage of donor islets and the need for long-term immuno-suppression. Recently, human stem cell-derived beta (SC-β) cells have been developed, providing a pathway to produce an unlimited supply of insulin-producing cells^12,13^. However, these cells still need to be immunoprotected or encapsulated to prevent the immune and autoimmune responses.

Cell encapsulation has indeed shown great promise in numerous animal studies. Among the different materials used for cell encapsulation, alginate is one of the most prevalent ones to date^14–16^, due in a large part to its mild gelation conditions and minimal toxicity^15–18^. However, foreign body reaction (FBR), a complex process involving protein adsorption, monocyte/granulocytes/macrophage adhesion, giant cell formation, and cross-talks between macrophages/giant cells and other immune/fibroblast cells, against alginate microcapsules is often observed and can be further elevated by encapsulated cells or xenogeneic donor tissue^5,19,20^. The cellular overgrowth (CO) and the fibrosis, an end result of the FBR^21,22^ that the body forms to isolate foreign implants reduce and even cut off the diffusion of nutrients and oxygen to the encapsulated cells, causing cell necrosis. To mitigate the CO of alginate microcapsules, investigators recently took an expensive, time-consuming but effective high throughput approach. Vegas et al. created a library of almost 800 chemically modified alginate derivatives and identified a few “hits” (e.g., Z1-Y15 containing triazole group) that effectively mitigated CO in mice and non-human primates^23,24^.

We report here a totally different, more rational and much less expensive approach to develop CO-mitigating, chemically modified alginates. Nonspecific protein adsorption onto implanted material is considered the first and critical step of FBR^25–27^. An antifouling material or surface that is highly resistant to protein adsorption and cell attachment is expected to suppress FBR and subsequently the CO and formation of fibrosis^25^. Recently, zwitterionic polymers, bearing zwitterions of carboxybetaine (CB), sulfobetaine (SB) and phosphorycholine, have been extensively studied in regards to their ultra-low-fouling properties^28–30^. For example, zwitterionic poly(carboxybetaine methacrylate) (PCBMA) hydrogels have been shown to resist the formation of fibrotic capsule for at least 3 months after subcutaneous implantation in mice^26^. Based on these previous studies, we rationalized that chemically modifying alginate with zwitterionic groups might lead to a different class of CO-mitigating alginate derivatives.

We first modify alginates (Ultrapure VLVG, SLG20, SLG100) with a zwitterionic group, SB and find that the modification reproducibly reduces the CO of the alginate microcapsules (diameter: 500~700 µm) in different species: C57BL/6J mice (intraperitoneal implantation), dogs (intraperitoneal) and pigs (omental pouch). To show the observed effect is reproducible, we have done a total of 17 mouse experiments with different types of alginates and different time points up to 6 months. Consistently, the SB-alginate microcapsules induce significantly less CO than the unmodified control and most of the times almost free of CO. Interestingly, the CO-mitigating effect of the zwitterionic modification is also observed in carboxybetaine-based alginates (i.e., CB-alginates). Additional experiments in large animals including dogs and pigs show similarly reduced CO of the SB-alginate compared to the unmodified SLG20 or SLG100, indicating the potential translatability of the zwitterionic modification. Then we encapsulate rat islets using either the SB-alginate microcapsules or unmodified control microcapsules and transplant them intraperitoneally in C57BL/6J mice with streptozotocin (STZ)-induced diabetes. The SB-alginate microcapsules result in significantly better long-term glycemic control, up to 200 days. Characterization of retrieved microcapsules and islets confirms the CO-mitigating property of the SB-alginate microcapsules as well as islet survival and function. Compared with the previously published high throughput approach, the zwitterionic modification represents a much simpler and less expensive strategy for the design and development of super-biocompatible alginates. We believe that these zwitterionically modified-alginates and our approach may contribute to a cell encapsulation therapy for T1D and potentially other hormone-deficient diseases in the future.

## Results

### Development of zwitterionically modified alginates

**Fig. 1:**
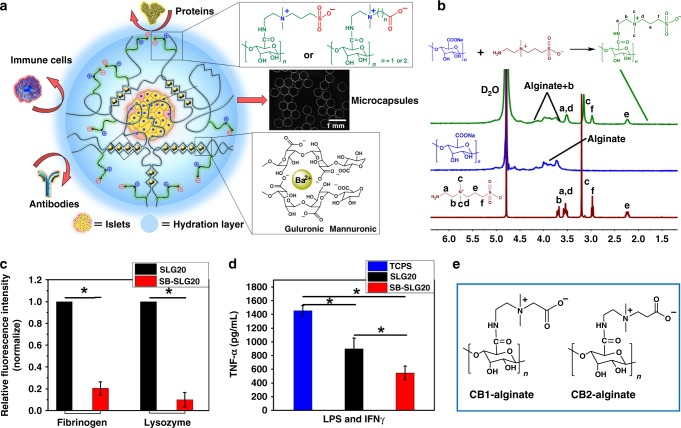
Design of zwitterionically modified alginates and their in vitro characterizations. **a** Schematic illustration of zwitterionically modified alginate microcapsules encapsulating islets. **b** Synthetic pathway and ^1^H NMR characterization of sulfobetaine (SB)-modification of alginate. **c** Adsorption of FITC-labeled fibrinogen and lysozyme on the surfaces of different alginate hydrogels quantified by ImageJ. Mean ± SEM; *n* = 6; *P < 0.05. **d** Quantification of TNF-α secretion from macrophages cultured on different surfaces. Mean ± SEM; *n* = 5; *P < 0.05. **e** Chemical structures of CB1-alginate and CB2-alginate conjugates

Recently, zwitterionic polymers and hydrogels have been extensively investigated due to their attractive ultra-low biofouling and biocompatible characteristics^28^. However, harsh required conditions such as UV irradiation or generation of free radical groups during the gelation of zwitterionic materials can be harmful to encapsulated cells, limiting broad biomedical applications^31–33^. We hypothesized that we could overcome this limitation but maintain the biocompatibility of zwitterionic compounds by developing a group of zwitterionically modified alginates. In these alginate derivatives, the zwitterionic moiety provides high surface hydration^34^, resistance to protein adsorption or cell adhesion, and mitigation of CO, while the alginate backbone remains crosslinkable with mild gelation condition and allows formation of microcapsules using an electrospraying technique (Fig. 1a).

Among zwitterionic groups, the SB group was firstly chosen in our studies because of its excellent antifouling performance, commercial availability and low cost^35,36^. In order to modify the alginate with SB group, we designed and synthesized SB-NH_2_ monomer according to Supplementary Fig. 1. Then, we chose low molecular weight (MW), ultrapure alginate VLVG as the starting material. 2-chloro-4, 6-dimethoxy-1, 3, 5-triazine (CDMT) and *N*-methylmorpholine (NMM) were used as coupling reagents to conjugate alginate with SB-NH_2_ via a triazine-based coupling reaction (Fig. 1b). The SB-based alginate conjugate was characterized by ^1^H NMR spectrum (Fig. 1b) where a peak at 3.20 ppm was attributed to the six protons in two methyl groups attached to the quaternary amine in the SB pendant group. The result suggested that SB-NH_2_ was successfully conjugated to alginate. About 30.5% modification degree of the starting alginate was confirmed by NMR data analysis. Using similar procedures, we also modified higher molecular weight alginates, SLG20 and SLG100.

To examine how the zwitterionic modifications may have affected the physiochemical properties of the alginates and microcapsules, we performed a number of characterizations. First, the surface roughness of SB-SLG20 and SLG20 microcapsules, assessed by atomic force microscope (AFM), were 11 ± 1 nm and 17 ± 15 nm, respectively (Supplementary Fig. 2). The SB-SLG20 capsules appeared slightly smoother but these two kinds of alginate capsules had no statistical difference in the surface roughness. We then evaluated whether this zwitterion modification changed the surface charge. Zeta-potentials of SLG20 and SB-SLG20 hydrogels were −17.3 ± 0.5 and −12.2 ± 0.3 mV, respectively (Supplementary Fig. 3). Zeta-potentials of SB-SLG20 and SLG20 hydrogels were similar and they are both negatively charged polymers. To compare the mass transfer of the unmodified and modified alginate hydrogels, we immersed SLG20 and SB-SLG20 hydrogels into different molecular weight, FITC-labeled dextran standards, respectively. The results (Supplementary Fig. 4) indicate that the diffusion rate of SB-SLG20 hydrogel was similar to that of SLG20 hydrogel regardless of molecular weight of dextrans. Since the mechanical property of the microcapsules is an important consideration in the success of cell encapsulation, it was evaluated in our studies by a Texture Analyzer. SB-SLG20 microcapsules under force were slightly stronger than SLG20 microcapsules (Supplementary Fig. 5). This might be attributed to the 40% SLG100 alginate (which has a larger molecular weight than SLG20) addition during preparation of the SB-SLG20 solution. Taken together, the zwitterionic modification did not seem to change the physiochemical properties of the microcapsules significantly.

Protein adsorption on the surface of an implanted medical device is the first step in a foreign body response, which will eventually affect the performances of the device^25,37^. Therefore, protein adsorption on the modified alginate was studied in our work, with unmodified SLG20 alginate as control. Two model proteins, fibrinogen (340 kDa, isoelectronic point: 5.5) and lysozyme (14 kDa, isoelectronic point: 11.1), were used to study the adsorption on the alginate hydrogel surfaces. These model proteins represent different molecular weights, structural stability, and isoelectronic points. Relative to SLG20 hydrogels, the amount of fibrinogen and lysozyme adsorptions on SB-SLG20 is 20.3 and 9.8%, respectively (Fig. 1c and Supplementary Fig. 6), indicating a strong resistance to non-specific protein adsorption. The excellent antifouling property of SB-SLG20 is probably due to the strong hydration of the SB groups^34^.

We then studied macrophage activation on the modified alginate hydrogels by seeding murine bone marrow derived macrophages (BMDM) and examining release of tumor necrosis factor-α (TNF-α) as a representative pro-inflammatory cytokine. After stimulation with lipopolysaccharide/interferon gamma (LPS/IFNγ) which is known to induce a pro-inflammatory macrophage phenotype^38^, the BMDMs cultured on the SB-SLG20 hydrogels secreted lower levels of TNF-α when compared to those cultured on the SLG20 hydrogels or tissue culture polystyrene plates (TCPS) (Fig. 1d). This study demonstrated that incorporating a zwitterionic moiety into alginate effectively inhibited the inflammatory activation of macrophages in vitro.

We also studied the impact of the alginate microcapsules on toll-like receptors (TLRs) signaling. TLRs are a class of proteins that play a key role in the innate immune system^39^. We used human embryonic kidney (HEK) cell line that expresses specific TLR signaling. The SLG20 and SB-SLG20 microcapsules (Supplementary Fig. 7) did not activate TLR2 or TLR4 but they did inhibit the signaling, indicating SLG20 and SB-SLG20 hydrogels were not immunostimulatory. More interestingly, SB-SLG20 capsules were shown to inhibit TLR2, more than SLG20 capsules and the control. These results again point to the potential anti-inflammatory effect of the zwitterionic modification.

To explore whether the in vitro anti-fouling and anti-inflammatory properties translate into CO mitigation in vivo, we performed a number of animal experiments. In addition to the SB-alginate, we also designed, synthesized and tested two other kinds of zwitterionically modified alginates (CB1-alginate and CB2-alginate, Fig. 1e; see Supplementary Figs. 8 and 9 for related NH_2_ terminated monomers CB1-NH_2_ and CB2-NH_2_).

### Zwitterionically modified alginates mitigate CO in mice

To evaluate the biocompatibility of SB-modified alginates, we first chose immunocompetent C57BL/6J mice because this strain was previously shown to elicit a strong CO against unmodified alginate microcapsules^40^. Microcapsules of SB-VLVG alginate were fabricated using electrospraying technique and had a uniform spherical morphology and diameters ranging from 450 to 550 μm, as shown in Fig. 1a and Supplementary Fig. 10. Unmodified SLG20 alginate was processed into microcapsules with similar size and morphology and used as control. The microcapsules were implanted in the intraperitoneal space of C57BL/6J mice and retrieved for characterization 14 days post implantation.

**Fig. 2:**
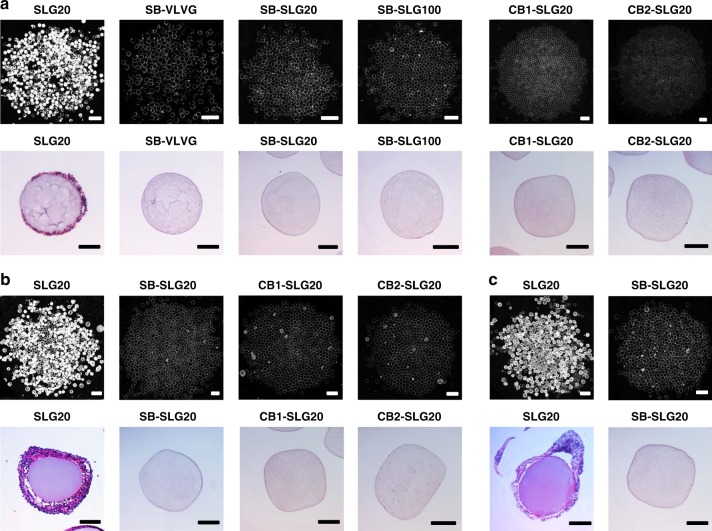
SB and CB modified alginates mitigate CO in mice. **a** Representative phase-contrast images of retrieved microcapsules made from different alginates (SLG20, *n* = 19, see Supplementary Fig. 11 for complete data; SB-VLVG, *n* = 10, see Supplementary Fig. 12 for complete data; SB-SLG20, *n* = 16, see Supplementary Fig. 14 for complete data; SB-SLG100, *n* = 10, see Supplementary Fig. 15 for complete data; CB1-SLG20, *n* = 5, see Supplementary Fig. 16a for complete data; CB2-SLG20, *n* = 5, see Supplementary Fig. 16b for complete data), 14 d post intraperitoneal implantation in C57BL/6J mice (Scale bar, 2000 μm) and corresponding H&E stained histological analysis (Scale bar, 200 μm). **b** Representative phase-contrast images of retrieved microcapsules, 100 d post intraperitoneal implantation in C57BL/6J mice (Scale bar, 2000 μm) and corresponding H&E stained histological analysis (Scale bar, 200 μm). (SLG20, *n* = 12, see Supplementary Fig. 17 for complete data; SB-SLG20, *n* = 10, see Supplementary Fig. 18 for complete data; CB1-SLG20, *n* = 4, see Supplementary Fig. Supplementary Fig. 20a for complete data; CB2-SLG20, *n* = 4, see Supplementary Fig. 20b for complete data). **c** Representative phase-contrast images of retrieved microcapsules, 180 d post intraperitoneal implantation in C57BL/6J mice (Scale bar, 2000 μm) and corresponding H&E stained histological analysis (Scale bar, 200 μm). (SLG20, *n* = 7, see Supplementary Fig. 22 for complete data; SB-SLG20, *n* = 7, Supplementary Fig. 21 for complete data)

To characterize the CO, we fused dark-field microscopic images of all retrieved microcapsules to obtain a composite view. In the images, the whiteness on the microcapsule surfaces (Fig. 2a) indicated the cellular deposition. Clearly, the control microcapsule (SLG20) as shown in Fig. 2a induced variable and substantial cellular deposition (see Supplementary Fig. 11 for all other 18 samples from 6 batches with a total *n* = 19), which was consistent with that of previously reported work^41^. In contrast, there was almost no cellular deposition observed on the SB-VLVG alginate microcapsules (Fig. 2a and see Supplementary Fig. 12 for all other 9 samples from three batches with a total *n* = 10). The representative H&E staining of microcapsule cross-sections (Fig. 2a) further confirmed that the surfaces of SB-VLVG alginate microcapsules were almost free of CO while the surfaces of SLG20 microcapsules had visible CO. To examine whether the observation was reproducible, another batch of SB-VLVG alginate was synthesized and evaluated for CO. This batch of retrieved SB-VLVG alginate as shown in Supplementary Fig. 12 also exhibited almost no cellular deposition. These results suggest that SB-VLVG alginate microcapsules mitigated CO effectively and reproducibly. This may be attributed to the zwitterionic SB group that increases surface hydration, reduces biofouling and improves biocompatibility of alginate. We have also compared directly one of the “hits” from the library screening^23^ with SB-VLVG alginates. As shown in Supplementary Fig. 13, there were minimal and similar levels of cellular depositions on our modified alginate and the one based on previous library screening method^23^, suggesting comparable biocompatibility despite different chemistries and approaches.

To verify that the CO-mitigating effect of SB modification does not depend on the type of alginate, we also synthesized SB-SLG20 alginate and SB-SLG100 alginate using the same method. Due to their higher molecular weights, the SLG20 (MW: 75–150 kDa) and SLG100 (MW: 150–250 kDa) form stronger hydrogels than VLVG alginate (MW < 75 kDa), and may have broader applications in biomedicine. The SB-SLG20 and SB-SLG100 alginate microcapsules were implanted in the intraperitoneal space of C57BL/6J mice and then retrieved after 14 days, with SLG20 microcapsules as control. Dark-field images of all retrieved SB-SLG20 alginate microcapsules (*n* = 16; Fig. 2a and all other samples from three batches in Supplementary Fig. 14) and SB-SLG100 microcapsules (*n* = 10; Fig. 2a and all other samples from two batches in Supplementary Fig. 15) showed very little CO. H&E staining (Fig. 2a) also indicated that there was almost no CO around retrieved SB-SLG20 and SB-SLG100 alginate microcapsules. In contrast, the conventional, unmodified SLG20 microcapsules induced varied degrees of CO, and some elicited severe CO. To explore whether other zwitterionic moieties play a similarly important role in mitigating CO, the biocompatibility of CB-alginates was evaluated. Interestingly, CB1-SLG20 and CB2-SLG20 (Fig. 2a and see Supplementary Fig. 16 for all other samples; *n* = 5 from one batch) microcapsules were also found to have little or no CO. Taken together, these results suggest that the CO-mitigation of zwitterionic modification is reproducible, and independent of alginate or zwitterion types, at least during 2-week intraperitoneal implantation in mice.

To investigate whether the modification can mitigate CO for a much longer term, we implanted SB-SLG 20 and CB-SLG20 microcapsules in C57BL/6J mice and retrieved them after 100 days. Retrieved SLG20 microcapsules (Fig. 2b and Supplementary Fig. 17, *n* = 12 from three batches) exhibited significant cellular deposition, which was further verified by histological analysis (Fig. 2b). Moreover, some of the retrieved SLG20 microcapsules even aggregated together, a sign of severe FBR (Supplementary Fig. 17). However, SB-SLG20 microcapsules (Fig. 2b and Supplementary Fig. 18, *n* = 10 from two batches) had a much lower level of cellular deposition, consistent with histology results (Fig. 2b). We also verified that SB-SLG100 microcapsules mitigated the CO effectively at 100 days (Supplementary Fig. 19, *n* = 4) indicating that this CO-mitigating zwitterionic modification is independent of alginate types for long-term implantation. Moreover, CB1-SLG20 and CB2-SLG20 microcapsules (Fig. 2b and Supplementary Fig. 20, *n* = 4) also had almost no CO. To further evaluate the longevity of the CO-resistant property, SB-SLG20 microcapsules were examined 180 days after implantation in C57BL/6J mice. As shown in Fig. 2c and Supplementary Fig. 21 (*n* = 7), SB-SLG20 microcapsules were largely free of cellular deposition after retrieval, which was consistent with H&E staining. However, there was severe CO observed on the surfaces of unmodified SLG20 microcapsules (Fig. 2c and Supplementary Fig. 22, *n* = 7). A striking difference between the modified and unmodified microcapsules was also observed in macroscopic photos of the retrieved samples (Supplementary Fig. 21b and 22b). The retrieved SLG20 microcapsules appeared mostly white in Petri dishes, indicating severe cellular deposition (Supplementary Fig. 22b, bottom two rows), while the near transparent appearance of the retrieved SB-SLG20 microcapsules suggested negligible CO (Supplementary Fig. 21b, bottom two rows).

**Fig. 3:**
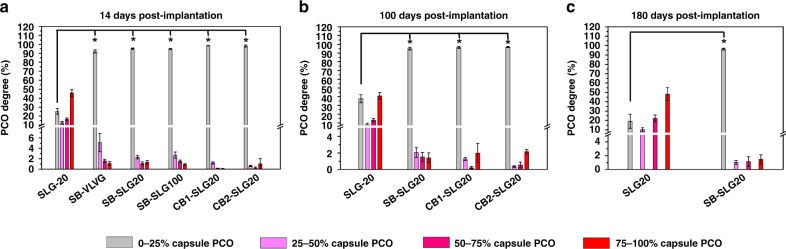
PCO evaluation of retrieved microcapsules. **a** Quantification of PCO for retrieved microcapsules, 14 d post implantation. Mean ± SEM; *n* = 19 for SLG20; *n* = 10 for SB-VLVG; *n* = 16 for SB-SLG20; *n* = 10 for SB-SLG100; *n* = 5 for CB1-SLG20; *n* = 5 for CB2-SLG20. *P < 0.05. **b** Quantification of PCO for retrieved microcapsules, 100 d post implantation. Mean ± SEM; *n* = 12 for SLG20; *n* = 10 for SB-SLG20; *n* = 4 for CB1-SLG20; *n* = 4 for CB2-SLG20. *P < 0.05. **c** Quantification of PCO for retrieved microcapsules, 180 d post implantation. Mean ± SEM; *n* = 7 for SLG20; *n* = 7 for SB-SLG20. *P < 0.05

To quantify the observations described above, we calculated retrieval rates (Supplementary Table 1) and categorized all retrieved microcapsules based on the percentage of surface coverage by “pericapsular cellular overgrowth” or PCO^24,42,43^: 0–25, 25–50, 50–75, and 75–100%. For SLG20 control microcapsules after 14 days implantation (Fig. 3a), the 0–25% PCO category (a sign of no or little CO) accounted for 24.5% of all retrieved microcapsules, and the 75–100% PCO category (a sign of severe CO) made up to 42.5%. In contrast, more than 90% of all retrieved zwitterionically modified microcapsules fell into the 0–25% PCO category, a significant improvement over conventional SLG20 microcapsules. Similarly, during long-term (100–180 days) studies (Figs. 3b, c), 90% of modified alginate microcapsules developed minimal CO (i.e. within the 0–25% PCO category), while almost half of SLG20 control microcapsules had severe CO (i.e., within the 75–100% PCO category). These quantifications suggest that zwitterionic SB and CB modifications substantially reduced CO of alginate microcapsules in the intraperitoneal space of C57BL/6J mice in both short (14 d) and long terms (100 d). More remarkably, zwitterionic SB-SLG20 was shown to mitigate the CO effectively, up to half a year.

Lastly, to better understand the phenotypes of adhered cells on the retrieved microcapsules and the innate immune response caused by zwitterion-modified and unmodified alginates, we implanted SLG20 and SB-SLG20 microcapsules in C57BL/6 mice for 2 weeks and immunologically analyzed the capsules and the intraperitoneal fluid surrounding the capsules. The retrieved capsules were stained by a number of cellular markers including α-smooth muscle actin (SMA), CD68, F4/80, CD11b, and Ly-6G/Ly-6C (Supplementary Figs. 23–26). These staining experiments revealed that the cells attached to microcapsules included monocytes, granulocytes, macrophages and fibroblasts, and there was a significant reduction of adhesion of these cells, particularly monocytes and neutrophils, after the zwitterionic modification consistent with the phase-contract images.

From immune profiling of the peritoneal fluid 2 weeks post implantation with 40 different cytokines (Supplementary Fig. 27), we observed interestingly that samples fabricated with modified alginate contained less inflammatory cytokines/components/chemokines in the intraperitoneal fluid than those with unmodified control, including C5/C5a, IP-10, TREM-1, IL-1β, IL-1a, CCL1, CCL2, CCL3^44,45^. TIMP-1 was also downregulated in the samples made with modified alginate, which inhibits matrix metalloproteinase and promotes fibrosis^46^. Another interesting observation was that the unmodified alginate samples contained CXCL1, CXCL2, CXCL12, which are powerful neutrophil chemoattractants that are involved in many immune responses including wound healing, cancer metastasis, and angiogenesis^47^. The results from immunostaining also verified the neutrophil trafficking in unmodified samples (Supplementary Fig. 26). Both modified and unmodified samples contained similar levels of chemokine CXCL13, which attracts B cells in peritoneum and promotes antibody production^48^. M-CSF (CSF1), secreted by macrophages and fibroblasts, is similarly expressed in both samples and important for the survival and proliferation of macrophages, confirming the local milieu of fibroblasts and macrophages^49^. IL-16, also expressed by both samples, is a lymphocyte chemoattractant factor for CD4^+^ lymphocytes, which not only regulates migration of all CD4^+^ T cells but also facilitates the expansion of CD4^+^ CD25^+^ Treg cells^50^. In summary, the immune profiling results seem to suggest that the zwitterionic modification influenced the cytokine expression in the intraperitoneal fluids surrounding the capsules and downregulated several inflammatory cytokines particularly neutrophil chemoattractants. More work will be needed in the future to fully understand the exact roles of all the cytokines we profiled in the host responses against the microcapsules.

### FBR mitigation in dogs and pigs

**Fig. 4:**
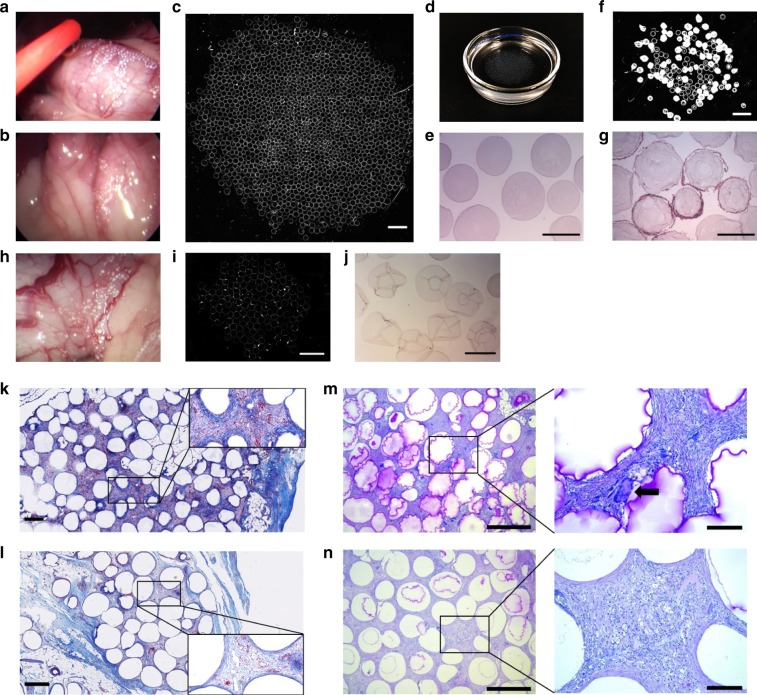
SB modified alginates mitigate FBR in dogs and pigs. **a** A laparoscopic image during retrieval of SB-SLG20 alginate microcapsules, 45 days after intraperitoneal implantation in a dog. **b** A laparoscopic image during retrieval of SLG20 control microcapsules. **c** A phase contrast image of retrieved SB-SLG20 microcapsules (*n* = 3; scale bar, 2 mm; see Supplementary Fig. 28 for complete data). **d** Retrieved SB-SLG20 microcapsules in a Petri dish. **e** H&E stained cross-sectional image of retrieved SB-SLG20 microcapsules (Scale bar, 500 μm). **f** A phase contrast image of retrieved SLG20 microcapsules (*n* = 1; scale bar, 2 mm). **g** H&E stained cross-sectional image of retrieved SLG20 microcapsules (Scale bar, 500 μm). **h** A laparoscopic image during retrieval of SB-SLG20 alginate microcapsules, 90 days after intraperitoneal implantation in a dog. **i** A phase contrast image of retrieved SB-SLG20 microcapsules (*n* = 2; scale bar, 2 mm; see Supplementary Fig. 29 for complete data). **j** H&E stained cross-sectional image of retrieved SB-SLG20 microcapsules (Scale bar, 500 μm). **k** Representative Masson’s trichrome staining (and a higher magnification) images of retrieved SLG100 alginate microcapsules (*n* = 2; scale bar, 500 μm), 1 month after implantation into the pig omental bursa. **l** Representative Masson’s trichrome (and a higher magnification) staining images of retrieved SB-SLG100 alginate microcapsules (*n* = 2; scale bar, 500 μm). **m** PAS-stained histology (and a higher magnification; scale bar, 200 μm) images of retrieved SLG100 microcapsules (Scale bar, 1 mm). Arrow indicates foreign body giant cells. **n** PAS-stained histology (and a higher magnification; scale bar, 200 μm) images of retrieved SB-SLG100 microcapsules (scale bar, 1 mm)

Next, we explored whether the observations in mice would translate to large animals such as dogs and pigs. First, SB-SLG20 microcapsules (~500 µm) were implanted intraperitoneally into Beagle dogs (*n* = 3) using a minimally invasive laparoscopic procedure. Efforts were made to spread out the microcapsules as much as possible. Unmodified SLG20 microcapsules were also implanted in one dog as control. The biocompatibility of the microcapsules was assessed 45 days after implantation using a similar laparoscopic procedure. There was no visible adhesion of SB-SLG20 microcapsules to host tissue (Fig. 4a), and they were easily dissociated from the implant site using either saline washing or catheter manipulation. A fraction of the microcapsules were aspirated out for characterization (Supplementary Movie 1). In contrast, the SLG20 microcapsules mostly adhered to the surrounding tissues and some were even fully embedded (Fig. 4b), making retrieval difficult. Multiple aspirations were needed to retrieve a sufficient number of microcapsules for characterization (Supplementary Movie 2). Dark-field microscopic images of retrieved SB-SLG20 microcapsules (Fig. 4c and Supplementary Fig. 28) revealed negligible cellular deposition, which was evidenced by the near transparent macroscopic appearance (Fig. 4d and Supplementary Fig. 28b). H&E stained section of retrieved capsules confirmed that there was minimal cellular deposition on the surfaces (Fig. 4e). In the contrast, the retrieved SLG20 microcapsules showed presence of strong CO (Fig. 4f) and many of them were covered with multiple cell layers (Fig. 4g). Moreover, we assessed the SB-SLG20 microcapsules again at 90 days post implantation from 2 of the 3 dogs that received implants. Still, the microcapsules had almost no tissue adhesion (Fig. 4h) and were mostly free of cellular deposition (Fig. 4i, j, and Supplementary Fig. 29).

To further evaluate the FBR to SB-alginate microcapsules, we chose insulin treated type 1 streptozotocin (STZ)-induced Göttingen minipigs and implanted SB-SLG100 microcapsules (Size: 500 ~ 700 μm) into pig omental bursa (*n* = 2), which is known to be extremely prone to elicit FBR following surgical intervention (Supplementary Fig. 30). In contrast to the laparoscopic implantation in dogs, the microcapsules were implanted as a whole without being spread out inside the omentum opening. (See Supplementary Fig. 30 for surgical details.) Unmodified SLG100 microcapsules were implanted as control (*n* = 2). One month after implantation, we excised the omentum and histologically analysed the microcapsules that were embedded. While both types of microcapsules caused FBR (as expected in such a fibrotic environment), there appeared to be differences. Unmodified SLG100 microcapsules had a dense and thick collagen deposition as indicated by the dark blue color with Masson’s trichrome staining and also induced a great number of inflammatory cells as indicated by the red color (Fig. 4k and Supplementary Fig. 31a for two pigs, respectively). On the contrary, SB-SLG100 microcapsules were observed to have a looser and thinner collagen deposition and were covered with a smaller number of inflammatory cells, as shown in Fig. 4l and Supplementary Fig. 31b. Moreover, periodic acid-schiff (PAS) staining of retrieved tissue showed that unmodified SLG100 microcapsules (Fig. 4m and Supplementary Fig. 32a) were generally associated with thicker and more mature bands of fibrous connective tissue, and had a marked FBR which included a chronic-active inflammatory cell infiltrate (lymphocytes and neutrophils), reactive fibroplasia, and foreign body giant cells (Fig. 4m, arrow). SB-SLG100 microcapsules (Fig. 4n and Supplementary Fig. 32b) had thinner and more wispy bands of connective tissue, and had a relatively reduced FBR including reduced fibroplasia, fewer chronic inflammatory cells (lymphocytes) and fewer/smaller multinucleated cells. H&E staining of cross-sections (Supplementary Fig. 33) confirmed that there was less cellular infiltration among the SB-SLG100 microcapsules compared with the SLG100 control. While more experiments with a larger n are required to perform quantitative, statistical comparisons, qualitatively the SB-alginate was shown to induce less FBR than the control even in a challenging, pro-fibrotic environment. All the results from mice, dogs, and pigs combined together point to the FBR or CO-mitigation effect of zwitterionic modifications for alginate microcapsules across species and at different implantation sites.

### Improvement of diabetes treatments in mice

**Fig. 5:**
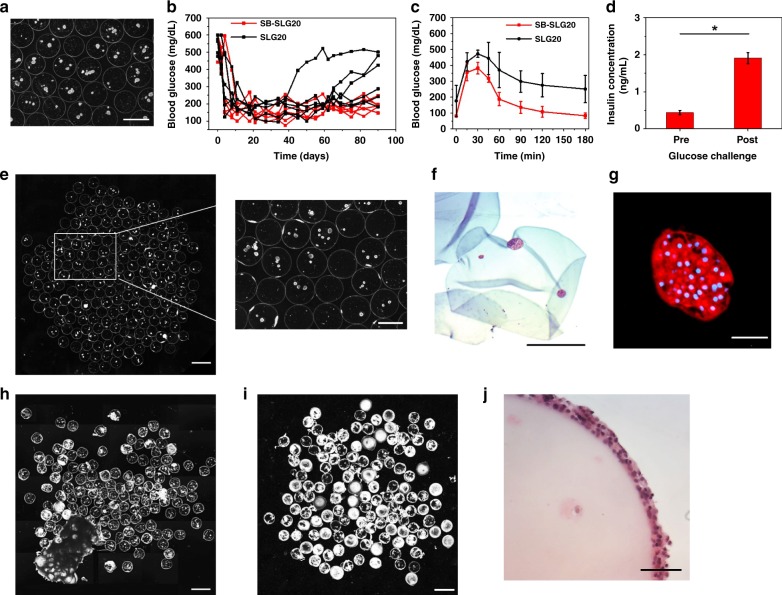
SB-SLG20 microcapsules improve diabetes correction in mice in a 90-day study. **a** A dark-field phase contrast image of SLG20 microcapsules encapsulating rat islets before transplantation. Scale bar, 1 mm. **b** Blood glucose concentrations of mice (*n* = 6 per treatment group). **c** Intraperitoneal glucose tolerance test (IPGTT) before retrieval (*n* = 3). **d** Ex vivo glucose-stimulated insulin secretion (GSIS) of retrieved islet-containing SB-SLG20 microcapsules, *n* = 3, Mean ± SEM, *P *<* 0.05. **e** A dark-field phase contrast image of retrieved islet-containing SB-SLG20 microcapsules (*n* = 6; see Supplementary Fig. 35 for complete data. Scale bars, 2 mm on the left and 1 mm on the right). **f** An H&E stained cross-sectional image of retrieved islet-containing SB-SLG20 microcapsules. Scale bar, 500 μm. **g** Immunohistochemical staining of a rat islet in a retrieved SB-SLG20 microcapsule. Insulin is stained red and nuclei are stained blue (Scale bar, 50 μm). **h**, **i** Dark-field phase contrast images of retrieved islet-containing SLG20 microcapsules from the normoglycemic mouse group (**h**) and failed ones (**i**). (*n* = 6; scale bar = 2 mm; see Supplementary Fig. 37 for complete data). **j** An H&E stained cross-sectional image of retrieved islet-containing SLG20 microcapsule (Scale bar, 200 μm)

After confirming that the zwitterionically modified alginates SB-SLG20 mitigated FBR in C57BL/6J mice and large animals, we explored its therapeutic potential as a cell encapsulation medium for treatment of T1D. SB-SLG20 microcapsules (Size: 800~1000 μm; the size distribution as shown in Supplementary Fig. 34) encapsulating rat islets (500 islet equivalents per mouse) were transplanted into the peritoneal cavity of streptozotocin (STZ)-induced C57BL/6J diabetic mice and evaluated for 90 days for their ability to restore normoglycemia. Rat islets were also encapsulated in unmodified SLG20 microcapsules as control (Fig. 5a). The BG level of the mice decreased to normal glycemic range (BG < 200 mg/dL) a few days after transplantation (Fig. 5b) for both groups. However, mice from the control group (i.e., unmodified microcapsules) experienced a shorter duration of glycemic control and four out of the six mice were unable to sustain normoglycemia within 90 days, whereas all the mice from the SB-SLG20 group remained normoglycemic for 3 months before the microcapsules were retrieved. We also performed an intraperitoneal glucose tolerance test (IPGTT) (Fig. 5c) 90 days after transplantation, immediately prior to retrieval on selected mice. While three mice with lowest BG levels from the SLG20 control group failed to reduce BG to normoglycemic range even 180 min after glucose challenge, mice in the SB-SLG20 group (*n* = 3) achieved normoglycemia within 90 min, confirming the improved function of transplanted islets (Fig. 5c). Furthermore, the glucose-stimulated insulin secretion (GSIS) assay performed on the retrieved SB-SLG20 microcapsules (Fig. 5d) showed that encapsulated islets were responsive to glucose change and secreted insulin, further supporting for normal islet function.

Post-retrieval characterizations also showed marked differences between the SB-SLG20 microcapsules and control microcapsules, with the former showing almost no cellular deposition (Fig. 5e and Supplementary Fig. 35). Over 90% of SB-SLG20 microcapsules fell into the 0–25% PCO category (Supplementary Fig. 36). In the SB-SLG20 microcapsules, there were numerous rat islets (See Fig. 5e and Supplementary Fig. 35 for all samples *n* = 6 from two batches) observed with healthy morphology (H&E staining in Fig. 5f) and positive insulin staining (Fig. 5g). On the contrary, retrieved SLG20 microcapsules showed a large variation in CO (See Fig. 5h, Fig. 5i and Supplementary Fig. 37 for all samples *n* = 6 from two batches). Approximately 19.1% of all retrieved SLG20 microcapsules were within the 0–25% PCO range and 48.3% fell into the 75–100% PCO category (Supplementary Fig. 36). Approximately 75% of the SLG20 microcapsules from the 2 normoglycemic mice had moderate to little CO, while the majority of the microcapsules from the 4 failed mice had severe cellular deposition, suggesting a correlation between CO level and diabetes correction. As expected, the islets in the microcapsules with cellular deposition either exhibited unhealthy morphology or were completely dead as shown by the H&E staining of cross-sections (Fig. 5j).

**Fig. 6:**
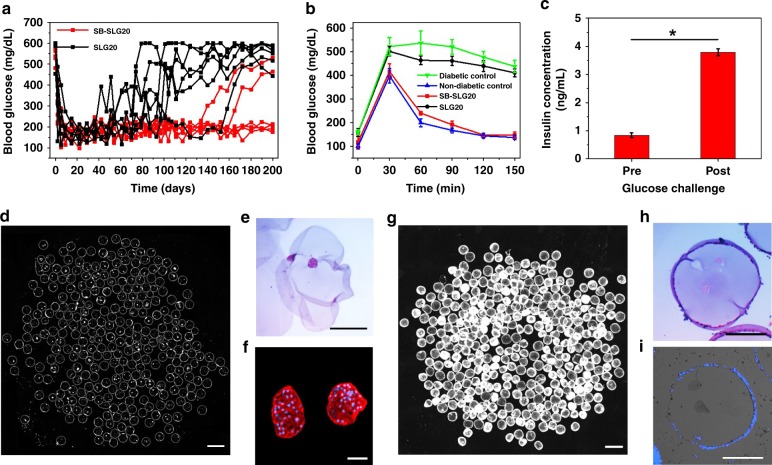
SB-SLG20 microcapsules improve diabetes correction in mice in a 200-day study. **a** Blood glucose concentrations of mice (*n* = 6 mice per treatment group). **b** Intraperitoneal glucose tolerance test (IPGTT) before retrieval (*n* = 3). **c** Ex vivo GSIS of the retrieved rat islets from SB-SLG20 microcapsules, *n* = 3, Mean ± SEM, *P < 0.05. **d** A dark-field phase contrast image of retrieved islet-containing SB-SLG20 microcapsules. (*n* = 6; scale bar, 2 mm; see Supplementary Fig. 38 for complete data). **e** An H&E stained cross-sectional image of retrieved islet-containing SB-SLG20 microcapsules. Scale bar, 500 μm. **f** Immunohistochemical staining of rat islets in retrieved SB-SLG20 microcapsules. Insulin is stained red and nuclei are stained blue (Scale bar: 50 μm). **g** A dark-field phase contrast image of retrieved islet-containing SLG20 microcapsules. (*n* = 6; scale bar, 2 mm; see Supplementary Fig. 40 for complete data). **h** An H&E stained cross-sectional image of retrieved islet-containing SLG20 microcapsules. Scale bar, 500 μm. **i** Immunohistochemical staining of rat islets in retrieved SLG20 microcapsules. Insulin staining is negative and nuclei are stained blue. Scale bar, 500 μm

To further study the robustness of the SB-alginate in improving islet encapsulation and sustaining normoglycemia, we performed a longer-term, 200-day transplantation experiment. Four out of the six diabetic mice transplanted with rat islets encapsulated in SB-SLG20 microcapsules maintained normoglycemia after 200 days (Fig. 6a); the shortest duration of glycemic control was ~135 days. However, almost all the mice transplanted with rat islets encapsulated in SLG20 microcapsules returned to hyperglycemia by 100 days after implantation. An IPGTT assay (Fig. 6b) 200 days after transplantation, right before retrieval showed that the mice (cured ones, *n* = 3) in the SB-SLG20 group cleared BG and restored normoglycemia at a rate comparable to that of non-diabetic mice, while the BG of the mice (*n* = 3) in the SLG20 control group failed to drop to normal range after 150 min, similar to non-transplanted diabetic mice. For the SB-SLG20 group, an ex vivo GSIS (Fig. 6c) of islets retrieved from cured mice (*n* = 3) indicated again the normal function of islets. Dark-field microscopic images of retrieved SB-SLG20 microcapsules (See Fig. 6d and Supplementary Fig. 38 for all samples *n* = 6 from two batches) from normoglycemic mice after 200 days revealed no or minimal cellular deposition on the microcapsules and the presence of numerous islets inside. PCO quantification showed that 81.5% of SB-SLG20 microcapsules were largely free of CO (Supplementary Fig. 39). More importantly, the retrieved islets were functional, as verified by H&E histological analysis (Fig. 6e) and positive insulin staining (Fig. 6f). In contrast, the SLG20 microcapsules produced severe CO as shown by dark-field phase contrast microscopic images (See Fig. 6g and Supplementary Fig. 40 for all samples *n* = 6 from two batches), by the PCO quantification (Supplementary Fig. 39), and by the H&E staining (Fig. 6h). The H&E staining also showed unhealthy or non-viable morphology of encapsulated islets and the immunohistochemical staining of insulin (Fig. 6i) was negative. Furthermore, the retrieval rate for the SB-SLG20 microcapsules was significantly higher than that for the SLG20 microcapsules (Supplementary Table 2). Taken together, the SB modification drastically improved the outcome of islet microencapsulation, achieving long-term glycemic correction for up to 200 days in STZ-treated C57BL/6J mice.

## Discussion

FBR against implanted biomaterials and medical devices represents a major hurdle to many biomedical engineering applications, particularly cell encapsulation. Unfortunately, despite its importance, FBR is an incompletely understood process involving complex biological cascades and high heterogeneity. For alginate microcapsules, which have been used for decades for cell encapsulation, it has been shown that many parameters including types of alginates^39,51^, purity of alginate (presence of endotoxins, proteins, and polyphenols)^39,52^, alginate compositions^53–56^, microcapsule size^41,57^ and subtle changes in formulations^58–62^ can all affect the degree of FBR or CO. Furthermore, the presence or absence of additional coating layers such as poly-L-lysine or chitosan resulted in varying degrees of CO^63,64^. The choice of cross-linking ions (usually calcium or barium) was reported to influence inflammatory response against alginate-based capsules^65^. Alginate capsules containing anti-inflammatory drugs has been employed as a strategy to mitigate the CO and improve the implantation outcome^66,67^. However, reproducibility of microcapsule performance even in mice has been a challenge for the field. Different labs often report different results in terms of CO^19,41,68–70^. Indeed, we have often observed in our laboratory variations of CO against the unmodified control microcapsules between experiments, among different animals in the same experiment, and even among different microcapsules within the same animal. In our present study, we made significant efforts to retrieve, image and analyze all microcapsules from all mice. For unmodified control microcapsules, while there always existed a small fraction that were relatively clean, the majority of them had CO to different degrees. These observations were consistent with those reported recently for microcapsules made of similar alginates with similar dimensions (<1mm)^41^. In contrast, for the zwitterionically modified alginates, we observed consistent and uniform reduction (and in some cases elimination) of CO. Additional large-animal experiments showed that the FBR-mitigating effect was reproducible across different animal species including C57BL/6J mice, dogs and pigs.

Although CO-mitigating, chemically modified alginates have been reported previously, those “hits” were discovered by time-consuming screenings of a total of 774 different types of chemical modifications^23^. Our zwitterionic modification of alginates represents a simpler and much less expensive approach and led to alginate derivatives that were shown comparable to those obtained by screening. The rationale is based on the well-studied anti-fouling properties of zwitterionic moieties. In this work, we also started to explore the mechanisms of the CO-mitigating effect. Our data, consistent with literature^34,71^, supported that zwitterionic groups due to their strong hydration mitigated nonspecific protein adsorption which is a key first step in foreign body responses. Likely as a result of the decreased protein adsorption, the zwitterionic modification altered macrophage activation, TLR2 inhibition and cytokine expression in the peritoneal fluid which might be contributing factors to the observed CO mitigation. Despite these studies, more work is required to elucidate the exact mechanisms.

To demonstrate and confirm the CO-mitigating effect of zwitterionic modification, we modified three different ultrapure, sterile alginates (VLVG, SLG20 and SLG100) and used three different zwitterions, a SB and two CBs. All the zwitterionically modified alginates (SB-alginates, CB1-alginate and CB2-alginate) were shown to mitigate CO compared to the unmodified control. Incorporating a zwitterionic moiety into alginate therefore opens up a new avenue for the design and development of super-biocompatible alginates.

The therapeutic potential of the SB modified alginate was explored through a type 1 diabetic mouse model using rat islets. Even in the presence of rat islets (i.e., xenogeneic tissue), the SB-SLG20 microcapsules had no or minimal CO in C57BL/6J mice, while the unmodified, control SLG20 microcapsules were mostly covered by CO. The reduced CO correlated with an improved outcome for islet encapsulation and transplantation. The SB-SLG20 microcapsules encapsulating rat islets enabled longer and more robust correction of STZ-induced diabetes in C57BL/6J mice. A 200-day cure was achieved for four out of six mice using SB-SLG20 microcapsules. Moreover, the ability of zwitterionic modification to mitigate FBR was translatable to higher-order species (dogs and pigs) and in different implantation sites (intraperitoneal cavity and omental bursa), suggesting great potential for future clinical applications. Zwitterionic materials have been used for a number of applications including fabrication of antifouling surfaces, grafting of implantable devices and biosensors, and formation of drug delivery micelles and nanogels^26,28,72–77^. Here, we report the use of zwitterionically modified materials for cell encapsulation for potential T1D treatment. This approach may contribute to a cell replacement therapy for not only T1D but also other hormone-deficient diseases such as hemophilia.

## Methods

### Study design

The aim of this study was to explore whether zwitterionically modified alginates could mitigate CO reproducibly at various implantation time points and across different species. To test this, all experiments using C57BL/6J mice, Sprague Dawley rats, and Beagle dogs were conducted at Cornell University, approved by the Cornell Institutional Animal Care and Use Committee, and carried out by trained personnel. Transplantation of alginate microcapsules in insulin treated type 1 STZ-induced Göttingen minipigs was performed at Novo Nordisk A/S, and the protocols were approved by the Danish Animal Experimentation Inspectorate and carried out by trained and licensed personnel. Alginate microcapsules were retrieved and imaged, and histopathology using H&E as well as special stains was performed by trained individuals.

### Materials/reagents

Di-*tert*-butyl dicarbonate, triethylamine, *N, N*-Dimethylethylenediamine, *β*-Propiolactone, *tert*-butyl bromoacetate, barium chloride, magnesium sulfate, magnesium chloride hexahydrate, phosphate-buffered saline (PBS; pH 7.4, 10 mM, 138 mM NaCl, 2.7 mM KCl), HEPES buffer, diethyl ether, ethyl alcohol, acetonitrile and dichloromethane (DCM) were obtained from Sigma-Aldrich. 2-chloro-4, 6-dimethoxy-1, 3, 5-triazine (CDMT), *N*-methylmorpholine (NMM), 1, 3-propanesultone and trifluoroacetic acid (TFA) were purchased from the Alfa Aesar. All the sodium alginates including VLVG (>60% G, 25 kDa MW), SLG20 (>60% G, 75–220 kDa MW), and SLG100 (>60% G, 200–300 kDa MW), were purchased from FMC BioPolymer Co. (Philadelphia, PA). Cyano-functionalized silica was purchased from SiliCycle. Rabbit anti-insulin antibodies (Cat. #ab63820) was purchased from Abcam, and Alexa Fluor 594-conjuaged donkey anti-rabbit igG (Cat. #A-21207) was purchased from Invitrogen. α-smooth muscle actin (SMA) (Cat. #C6198) was purchased from Sigma-Aldrich, and anti-mouse CD68 (Cat. #137012) was purchased from BioLegend. Anti-mouse F4/80 (Cat. MF48000) was purchased from ThermoFisher, and anti-CD11b (Cat. #ab133357) was purchased from Abcam. Anti-mouse Ly-6G/Ly-6C (Cat. #108419) was purchased from BioLegend. Proteome profiler array kit (Mouse Cytokine Array Panel A; Cat. #ARY006) was purchased from R&D Systems.

### Synthesis of SB-based conjugates

Synthesis of SB-NH_2_ material is shown in Supplementary Fig. 1. Briefly, di-*tert*-butyl dicarbonate (10.0 g, 45.8 mmol) and triethylamine (12.8 mL, 91.6 mmol) were added dropwise over 0.5 h to a solution of *N, N*-Dimethylethylenediamine (4.04 g, 45.8 mmol) in anhydrous ethyl alcohol (150 mL) at 0 °C. The mixture was stirred for 1 h at 0 °C and then for 18 h at room temperature. The white precipitate was filtered off and the filtrate was evaporated to obtain residue. The residue was dissolved in dichloromethane (150 mL), and the solution was washed successively with water. The organic layer was dried over anhydrous magnesium sulfate and evaporated to get *N, N*-dimethyl-2-((pivaloyloxy) amino) ethan-1-amine. ^1^H NMR (CDCl_3_, 400 MHz): δ 3.22 (t, 2H), 2.29 (t, 2H), 2.22 (s, 6H), 1.43 (s, 9H).

*N, N*-dimethyl-2-((pivaloyloxy) amino) ethan-1-amine (7.52 g, 40.0 mmol), 1, 3-propanesultone (4.9 g, 40.0 mmol) and acetonitrile (150 mL) were added into a 300 mL round-bottom flask. The mixture was stirred under nitrogen atmosphere for 48 h at 40 °C. After reaction, the solvent was removed by rotary evaporator. The product was precipitated by anhydrous diethyl ether and washed with anhydrous diethyl ether to get white powder. ^1^H NMR (D_2_O, 400 MHz): δ 3.41–3.63 (m, 6H), 3.17 (s, 6H), 2.98 (m, 2H), 2.24 (m, 2H), 1.43 (s, 9H).

Finally, 10.0 g of the obtained product was treated with a mixture of 20 mL trifluoroacetic acid (TFA) and 20 mL dichloromethane overnight at room temperature, concentrated with rotary evaporator, precipitated in anhydrous diethyl ether, and re-dissolved in DI water. Ion-exchange resin (Amberlyst A26, OH-form) was added into the solution for complete neutralization. The residue was lyophilized by freeze dryer to obtain product (SB-NH_2_ material). ^1^H NMR (D_2_O, 400 MHz): δ 3.70 (t, 2H), 3.54 (m, 4H), 3.20 (s, 6H), 2.97 (t, 2H), 2.24 (m, 2H).

Synthesis of SB-modified alginate: 0.5 g of VLVG alginate, SLG20 alginate or SLG100 alginate was soluble in 50 mL mixture solvent (40 mL of DI water and 10 mL acetonitrile). 225 mg of 2-chloro-4, 6-dimethoxy-1, 3, 5-triazine (CDMT) and 280 μL of *N*-methylmorpholine (NMM) were added. Then 0.36 g of SB-NH_2_ material was dissolved in 10 mL DI water and added into the mixture. The reaction was stirred overnight at 60 °C. The solvent was removed under reduced pressure and the solid product was redissolved in DI water. The solution was filtered through a pad of cyano-functionalized silica. It was then dialyzed against a 10,000 MWCO membrane in DI water for three days. Finally, the water was removed by freeze dryer to obtain SB-modified alginate. ^1^H NMR (D_2_O, 400 MHz): δ 3.5–5.3 (m, alginate protons), 3.87 (m, 2H), 3.61 (m, 4H), 3.14 (s, 6H), 2.91 (t, 2H), 2.20 (m, 2H). There was about 30.5% modification degree of the starting alginate through NMR analysis.

### Synthesis of CB-based conjugates

Synthesis of CB1-NH_2_ materials is shown in Supplementary Fig. 8. Briefly, *N, N*-dimethyl-2-((pivaloyloxy)amino)ethan-1-amine (7.52 g, 40.0 mmol), *tert*-butyl bromoacetate (7.8 g, 40.0 mmol) and acetonitrile (150 mL) were added into a 250 mL round-bottom flask. The mixture was stirred under nitrogen atmosphere for 48 h at 40 °C. After reaction, the solvent was removed by rotary evaporator. The product was precipitated by anhydrous diethyl ether and washed with anhydrous diethyl ether three times to get white powder. ^1^H NMR (D_2_O, 400 MHz): δ 4.21 (s, 2H), 3.72 (m, 2H), 3.57 (m, 2H), 3.31 (s, 6H), 1.32–1.54 (s, 18H).

Finally, 5.0 g of the obtained product was treated with a mixture of 20 mL trifluoroacetic acid and 20 mL dichloromethane overnight at room temperature, concentrated with rotary evaporator, precipitated in anhydrous diethyl ether, and redissolved in DI water. Ion-exchange resin (Amberlyst A26, OH-form) was added into the solution for complete neutralization. The residue was lyophilized by freeze dryer to obtain product (CB1-NH_2_ monomer). ^1^H NMR (D_2_O, 400 MHz): δ 4.31 (s, 2H), 3.99 (t, 2H), 3.55 (t, 2H), 3.36 (s, 6H). The CB2-NH_2_ monomer as shown in Supplementary Fig. 9 was synthesized using the same procedure as that for CB-1-NH_2_ monomer. ^1^H NMR of CB2-NH_2_ (D_2_O, 400 MHz): δ 3.63–3.75 (m, 4H), 3.53 (m, 2H), 3.18 (s, 6H), 2.96 (t, 2H).

Synthesis of CB-modified alginates: The CB1-alginate and CB2-algiante conjugates were synthesized using the same procedure as that for SB-alginate conjugate. The chemical structures of these CB-based alginate conjugates were confirmed by NMR. ^1^H NMR of CB1-alginate: *δ* = 3.5–5.3 (m, alginate protons), 3.84–4.10 (m, 4H), 3.72 (m, 2H), 3.34 (s, 6H). There was about 33.1% modification degree of the starting alginate through the NMR analysis. ^1^H NMR of CB2-alginate: *δ* = 3.5–5.3 (m, alginate protons), 3.87–4.06 (m, 4H), 3.80 (m, 2H), 3.39 (s, 6H), 2.95 (m, 2H). There was about 24.7% modification degree of the starting alginate through NMR analysis.

### Preparation of SB-based or CB-based alginate microcapsules

All the buffers were sterilized, and alginate solutions were filtered using a 0.2 μm filter before use. 2% (w/v) alginate (VLVG, SLG20, or SLG100) was dissolved in saline solution to prepare an alginate solution. 2% (w/v) SB-based alginate conjugate was dissolved in saline solution. The mixture of 60% (by volume) SB-based alginate solution and 40% (by volume) SLG100 solution were blended to obtain the SB-alginate solution. The CB-alginate solution was prepared using the same procedure.

Alginate hydrogel microcapsules were made using a custom-built electrospraying setup. Briefly, a high voltage power generator was connected to a blunt-tipped needle. This needle was attached to a 3 mL syringe containing alginate solution clipped to a vertically oriented syringe pump. Various sizes of microcapsules were prepared using different voltage, flow rate and needle gauges; 500–700 µm microcapsules were prepared using a voltage of 8–9 kV, a 0.2 mL/min flow rate and a 25-gauge blunt needle (SAI Infusion Technologies), 800–1000 µm microcapsules were prepared using a voltage of 6–7 kV, a 0.2 mL/min flow rate and a 20-gauge blunt needle (SAI Infusion Technologies). Alginate solution was sprayed into a sterile, grounded dish containing a 20 mM barium chloride solution. Once the alginate microcapsules were fabricated, they were collected immediately and washed with prepared HEPES buffer [NaCl 15.43 g, KCl 0.70 g, MgCl_2_•6H_2_O 0.49 g, 50 mL of HEPES (1 M) buffer solution in 2 L of DI water] three times and then washed additional two times with saline and kept at 4 °C before use.

### Protein adsorption

Two percent of SB modified alginate solution and two percent of SLG100 alginate solution were mixed at a 60:40 (v/v) ratio. Two percent of SLG20 was used as control. 0.2 mL of the above alginate solutions were added to a 24-well plate. Then, 0.2 mL of barium chloride solution (20 mM) as a cross-linking buffer was slowly added on the top of the gel. After 1 h solidification, these alginate hydrogels were removed from plates and washed with PBS five times before use.

The above alginate hydrogels were immersed in FITC-labeled fibrinogen solution (0.1 mg/mL in PBS) or FITC-labeled lysozyme solution (0.1 mg/mL in PBS) at room temperature for 1 h to allow protein adsorption on the hydrogel surfaces. Hydrogels were then gently washed with PBS five times to remove the unbound protein molecules. Fluorescence images of hydrogel surfaces were taken using a fluorescence microscope with 10× lens at a fixed exposure time. To ensure that all hydrogel samples were focused on the same plane, all images were taken at the edge of the hydrogels. The fluorescent intensity was quantified and analyzed using ImageJ software.

### Mechanical strength of microcapsules

The mechanical properties of different alginate microcapsules was tested with a Texture Analyzer XT plus (Stable Micro Systems, Godalming, UK) equipped with a force transducer (Resolution: 1 mN). Microcapsules were carefully monitored under a dissection microscope (Bausch and Lomb BVB-125). Uniaxial compression tests were performed as follows: a mobile probe (P/25L) with pretest speed of 0.5 mm/s, a test speed of 0.01 mm/s, and a post-test speed of 2 mm/s was used to compress individual microcapsules (*n* = 10) with a trigger force of two grams. The force (grams) was quantified at a 60% compression of the sample. Resulting measurements were analyzed via Texture Exponent software (v6.0).

### Mass transfer studies

To measure the diffusion rate of the unmodified and modified alginate hydrogels, we immersed SLG20 and SB-SLG20 hydrogels into three different saline solutions containing 10, 70, and 250 kDa FITC-labeled dextrans (1 mg/mL), respectively. The fluorescent intensity of the alginate hydrogel was analyzed using confocal microscopy.

### Surface roughness of capsules

Single capsules were carefully selected using a dissection microscope (Leica MZ75 microsystems), washed and placed on microscopic slides. The surface roughness of different microcapsules was determined by a Bruker Catalist atomic force microscope (AFM) with a Bruker DNP silicon nitride cantilever. Topographic imaging of the capsules was performed at room temperature using the contact mode. Surface roughness was calculated by using the NanoScope Analysis 1.8 software.

### Zeta potential measurement

Unmodified and modified alginate nanogels were prepared via a surfactant-free and organic solvent-free method^78^. Briefly, sodium alginate was mixed with ionic calcium by in situ crosslinking under ultrasound, and alginate nanogels were obtained. Zeta potentials of alginate nanogels were then investigated using Zetasizer Nano ZS (Malvern, U.K.). The value was recorded as the mean of five measurements.

### TLR signaling

To study whether zwitterionic modified alginate alters TLR signaling, we performed the HEK-Blue^TM^ TLR cell-based assays (InvivoGen, France) according to the manufacturer’s protocol. These HEK-Blue^TM^ cells express the Soluble Embryonic Alkaline Phosphatase (SEAP) gene which can be quantified using Quantiblue (InvivoGen, France)^39,79^. The SEAP gene is under the control of the NFκB/AP-1 responsive promoter. The information of cell lines, antibiotics, and concentration of agonists used to activate TLR signaling were shown in Supplementary Table 3. All cell lines were cultured in DMEM culture media (Lonza, Basel, Switzerland) supplemented with 10% de-complemented Fetal Calf serum, 50 U/ml Penicillin (Sigma, St. Louis, MO, USA), 50 μg/ml Streptomycin (Sigma, St. Louis, MO, USA) and 100 μg/ml Normocin (InvivoGen, Toulouse, France). HEK- Blue^TM^ cell lines for TLR2 expressing cells at a density of 2.8 × 10^5^ cells/ml and HEK- Blue^TM^ cell lines for TLR4 expressing cells at a density of 1.4 × 10^5^ cells/ml, were seeded in 96-well plates at 180 µl/well, respectively. After overnight culture, Cells were treated with 25 alginate capsules or TLR agonists as control to study whether alginate capsules can activate TLR signaling (Supplementary Table 3). Inhibition of TLR was studied by exposing HEK-Blue^TM^ cells with 25 alginate capsules for 1 h, followed by treatment with TLR agonists (Supplementary Table 3). QUANTI-Blue™ medium (InvivoGen, Toulouse, France) change to a purple-blue color in the presence of SEAP. Media supernatant from the stimulated cell-lines was mixed with QUANTI-Blue™ medium in a ratio of 1:10 (v/v). Activation of NFκB was finally quantified at 655 nm using a Versa Max ELISA plate reader (Molecular devices, Sunnyvale, CA, USA). The assay was performed with six repeats.

### Cytokine secretion

All animal procedures were approved by the Institutional Animal Care and Use Committee at the University of California Irvine prior to initiation of the study. BMDM were harvested from the femurs or tibia of 6–8 week-old C57BL/6J mice (Jackson Laboratories). Cells were treated with ACK lysis buffer (Invitrogen), centrifuged, and resuspended in D-10 media. D-10 media consists of Dulbecco’s modified Eagle medium (DMEM), 10% heat-inactivated FBS, 2 mM L-glutamine, 100 units per mL penicillin–streptomycin, and 10% conditioned media from CMG 14–12 cells expressing macrophage colony stimulating factor (M-CSF)^80^. After culture for one week, BMDM were dissociated using cell dissociation buffer (Invitrogen), and seeded on the tissue culture plates at a cell density of 100,000 cells/cm^2^ (Olympus Plastics) in fresh D-10 culture media. At 6 h culture after cell seeding, 10% of 500 μm various alginate capsules (v/v) were added and stimulated with a combination of 0.3 ng/mL lipopolysaccharide (LPS) and 1.0 ng/mL inter-feron gamma (IFNγ). After 12 h of incubation, the supernatants were collected and analyzed for TNF-α secretion by enzyme-linked immunosorbent assay (ELISA) following the manufacturer’s instructions (BioLegend).

### Rat islet isolation, purification, and encapsulation

Male Sprague-Dawley rats from Charles River Laboratories weighing about 300 g were used for harvesting islets. All rats were anesthetized using 3% isoflurane in oxygen throughout the whole procedure. Isolation surgeries were performed following by Lacy and Kostianovsky^81^. Briefly, the port vein was clamped and rat bile duct was cannulated. The pancreas was distended by an in vivo injection of cold 0.15% Liberase (Research Grade, Roche) in RPMI 1640 media solution. The perfused pancreatic organs were removed and put into 50 mL conical tubes on ice until the end of all surgeries. All the tubes containing the pancreas were then placed into in a 37 °C water bath for a 29 min digestion. After that, the digestion was quenched by adding cold M199 media containing 10% heat-inactivated fetal bovine serum (HIFBS) and lightly shaking. Digested pancreases were washed twice by the same aforementioned M199 media, filtered through a 450 mm sieve, and then suspended in a Histopaque 1077 (Sigma)/M199 media gradient and centrifuged at 1700 RCF at 4 °C. This gradient centrifugation step was repeated for higher purity islets. Finally, these islets were further isolated by a series of six gravity sedimentations, in which each supernatant was discarded after 4 min of settling. Purified islets were handpicked under the microscope and washed by sterile saline solution. Islets were then cultured overnight in RPMI 1640 media with 10% HIFBS and 1% penicillin/streptomycin for further use.

Prior to islet encapsulation, the cultured islets were centrifuged at 562 RCF for 1 min and washed with saline solution. After washing, islets were centrifuged again and all supernatant was removed. The islet pellet was then resuspended in a 2% alginate solution at an islet density of 500 islets. 800–1000 μm alginate microcapsules containing islets were crosslinked using a BaCl_2_ gelling solution and their sizes of microcapsules were controlled using similar procedures as the empty spheres (described above). After crosslinking, microcapsules with encapsulated islets were immediately washed five times with saline solution to remove residual BaCl_2_ gelling solution, transferred into corresponding cell culture medium, and cultured 4 h at 37 °C prior to implantation. Since the rat islets had variable sizes (50–300 µm) and also there was an inevitable loss of islets during the process of encapsulation, the total number of encapsulated islets was converted into islet equivalents (IE, normalized to 150 µm size) following a previously published method^82^.

### Implantation and retrieval in mice

Immuno-competent male C57BL/6J mice were used for transplantation. To create insulin-dependent diabetic mice, healthy C57BL/6J mice were treated (50 mg/kg mouse) with freshly prepared streptozocin (STZ) (Sigma Aldrich) solution (7.5 mg/mL in sodium citrate buffer solution) for 5 consecutive days. The BG levels of all the mice were monitored before transplantation. The mice whose non-fasted BG levels of the mice were above 300 mg/dL, were considered to be diabetic. All the mice were anesthetized using 3% isoflurane in oxygen and maintained at the same rate throughout the procedure. Prior to transplantation, all mice were injected a 0.05 mg/kg dose of buprenorphine subcutaneously as a pre-surgical analgesic, and their abdomens were shaved and sterilized using betadine and isopropyl alcohol. A 0.5 mm incision was made along the midline of the abdomen and the peritoneal lining was exposed using blunt dissection. The peritoneal wall was then grasped with forceps, and a 1-mm incision was made along the linea alba. A desired volume of empty microcapsules or islets-containing microcapsules loaded into a sterile pipette were implanted into the peritoneal cavity through the incision. The incision was closed using 5–0 taper tipped polydioxanone (PDS II) absorbable sutures. The skin was then closed over the incision using a wound clip.

The empty microcapsules (or with encapsulated rat islets) were retrieved from mice at desired time points post-implantation. Mice were euthanized by CO_2_ administration followed by cervical dislocation, and larger (∼3 mm) incisions were made in the skin and the peritoneum. 100 mM CaCl_2_ solution was injected into the abdominal cavity repeatedly and washed out all material microcapsules from the abdomen. In certain instances, the microcapsules needed to be retrieved manually if fibrosed directly to intraperitoneal tissues. The retrieved microcapsules were then submerged into a cross-linking solution containing 100 mM CaCl_2_ for ∼1 min (It is noted that the size of capsules became slightly smaller after retrieval especially for long-term studies possibly due to change of environment from body fluid to a Ca^2+^-containing stabilizing buffer that was used to collect the microcapsules), and transferred into 4% paraformaldehyde for fixation or cell culture medium. Lastly, the microcapsules were collected and stacked together into Histogel for histological sectioning.

### Implantation and retrieval in dogs

Dogs (Beagle dogs from Marshall Bioresources, Clyde, NY) were premedicated with gylcopyrolate and butorphanol, induced with propofol, and anesthetized using isoflurane and oxygen throughout the surgery. The abdomen was shaved and prepared for sterile surgery. A 10-mm laparoscopic camera port and two 5-mm instrument ports were percutaneously inserted into the abdomen. The abdomen was insufflated to 12 mm Hg pressure with CO_2_. Prior to transplantation, about 10 mL of prepared microcapsules (size: 500 μm) and 30 mL saline solution were loaded into the 60 mL sterilized syringe. This syringe was then connected with one side of 16-gauge sterile catheter. This flexible catheter was inserted into the left-side instrument port and used to distribute the capsules throughout the abdominal cavity. The alginate capsule solution was gently infused at a rate no greater than 30 mL/min. A laparoscopic probe was inserted through the right-sided 5-mm port. After implantation, the abdomen was then desufflated and the remaining parts were removed. The port sites were closed with 3–0 polydioxinone suture material and tissue glue.

The retrieval procedure of microcapsules was conducted using the similar method described for implantation. The implanted capsules were firstly located and photographed. About 100 mL of sterile pre-warmed saline solution was injected to gently wash the peritoneal cavity and then gently aspirated back into the syringe. Closure was conducted with the same method described for implantation.

### Implantation and retrieval in pigs

Four female Göttingen minipigs (Ellegaard Minipigs, Dalmose, Denmark) were acclimatized for three weeks. Following acclimatization, the animals were given 60 mg/kg streptozotocin on three consecutive days to induce a type 1 diabetic phenotype. The animals were subsequently titrated in on long acting exogenous insulin using QD dosing to achieve a target a plasma glucose ~10–15 mM. The animals were anaesthetized and underwent a surgical laparotomy performed lege artis. Briefly, the animals were placed in the dorsal recumbency and the surgical area prepared aseptically, a 6 cm incision was made from processus xiphoideus and extended the in the caudal direction. The stomach was identified and the greater omentum (bursa omentalis) was advanced to the incision site. The visceral and parietal sheet of the omentum was separate and a 3 cm incision was made in the parietal sheet. Approximately 30, 000 microcapsules (capsule size: 500–700 μm) suspended in 50 mL of sterile saline solution were administered into the omentum. The surgical incision was closed lege artis and the pigs were provided with antibiotic prophylaxis. After 1-month implantation, the pigs were euthanized with a lethal overdose of euthanal, a full necropsy was performed and omentum with embeded microcapsules extracted for histopathology.

### Imaging of the retrieved alginate microcapsules

For phase contrast imaging, retrieved alginate microcapsules were gently washed using saline solution and transferred into Petri dishes for phase contrast microscopy using an EVOS AMF4300 imaging system.

### Blood glucose monitoring

Starting post-transplantation, a small drop of blood was collected from the tail vein of each mouse using a lancet and tested using a commercial glucometer (Clarity One, Clarity Diagnostic Test Group), approximately three times a week. Mice with unfasted glucose levels below 200 mg/dL were considered normoglycemic.

### IPGTT assay

Prior to retrieval, glucose tolerance tests were performed to assess metabolic capacity. Mice were fasted overnight before an i.p. injection of glucose solution (2 g of glucose per 1 kg of body mass). BG levels were monitored at predetermined time points (0, 15, 30, 60, 90, 120, and 180 min) after injection.

### In vitro GSIS assay

Krebs Ringer Bicarbonate (KRB) buffer [98.5 mM NaCl, 4.9 mM KCl, 2.6 mM CaCl_2_, 1.2 mM MgSO_4_·7H_2_O, 1.2 mM KH_2_PO_4_ and 25.9 mM NaHCO_3_ (all from Sigma-Aldrich) supplemented with 20 mM HEPES and 0.1%% BSA (Serological)] was prepared beforehand. Encapsulated islets were then incubated for 60 min with 2.8 mM or 16.7 mM d-glucose under the same condition. The supernatants were collected, and insulin content was quantified using ultrasensitive mouse/rat insulin ELISA kit (ALPCO) with measurement by Synergy 4 Fluorescence Absorbance Microplate Reader (BioTek) at 450 nm wavelengths. All of the ELISA results were normalized to the IEQs.

### Cytokine profiling analysis

Semi-quantitative analysis of cytokine/chemokine levels was performed using Proteome Profiler^TM^Array, Mouse Cytokine Array Panel A (ARY006; R&D Systems) according to manufacturer’s instructions. Briefly, 500 μl of intraperitoneal liquid was extracted directly after injection of 1 ml PBS with proteinase inhibitor. For each membrane, 500 μl of protein solution was mixed with 500 μl of sample buffer (array buffer 4) and 500 μl of block buffer (array buffer 6). Fifteen mocroliters of reconstituted Mouse Cytokine Array Panel A Detection Antibody Cocktail was then added and incubated at room temperature for 1 h. The array membrane was incubated with block buffer (array buffer 6) for 1 h on a rocking platform shaker. The tray should be oriented to ensure that each membrane rocks end to end in its well. The block buffer was then aspirated, and the prepared sample/antibody mixture was added onto the membrane and incubated overnight at room temperature on a rocking platform shaker. Each membrane was washed three times with 20 ml of 1X wash buffer for 10 min on a rocking platform shaker. Membranes were incubated in 1.5 ml of streptavidin-HRP (1:1,000 dilution) in array buffer 5 for 30 min at room temperature on a rocking platform shaker, and then washed with wash buffer three more times. Antibody-antigen complexes were visualized and analyzed using LI-COR C-Digit.

### Histological analysis and immunostaining

The retrieval microcapsules were fixed in 4% paraformaldehyde, stacked together into Histogel, embedded in paraffin and then sectioned by Cornell Histology Core Facility. The samples were sliced on a microtome at a thickness of 5 μm. Paraffin sections were then stained with hematoxylin/eosin.

To conduct immunofluorescence staining of retrieved islets, the histological slides were deparaffinized by sequential washing in xylene, 100, 90, and 75% ethanol, and DI water. These slides were then boiled in 1 mM EDTA for antigen exposure. Non-specific binding was blocked with 10% goat serum for 45 min at room temperature. After blocking, slides were decanted and incubated with primary rabbit anti-insulin antibodies (1:200) overnight at 4 °C. The sections were then washed and incubated with the FITC-conjugated secondary antibodies, 594-conjuaged donkey anti-rabbit igG (1:400 dilution) for 30 min at room temperature. Slides were washed twice with water, labeled with DAPI, and covered with coverslips. Fluorescence images were captured using an EVOS AMF4300 imaging system.

To conduct immunofluorescence staining of retrieved alginate microcapsules, samples were fixed in 4% paraformaldehyde overnight before use. Samples were washed with Krebs Buffer three times. Samples were then washed with PBS three times and a 1% Triton X-100 solution was used to permeabilize cells. Samples were incubated for 15 min at room temperature. Samples were then transferred into 1% albumin solution for 30 min at room temperature. Next, the microcapsules were incubated for 1 h in an immunostaining cocktail solution consisting two drops of DAPI and specific antibodies (1:200) in 1% albumin solution. Staining solution was then removed and washed twice with PBS solution containing 0.1% tween 20. Samples were washed twice with PBS and then transferred into a concave glass slide. Excess PBS was removed, and 50% glycerol solution was added. The samples were finally covered with coverslips. Fluorescence Images were captured under a Zeiss LSM710 confocal microscope at Cornell Biotechnology Resource Center Imaging Facility.

### Evaluation of PCO

The retrieved microcapsules were evaluated using phase-contrast microscopy to determine the degree of CO following the previous method^43^. For retrieved microcapsules, the degree of CO was determined by categorizing coverage percentage of surface area that was covered by PCO: 0–25, 25–50, 50–75, 75–100%. The PCO degree for retrieved capsules was calculated using the equation: PCO degree (%) = (the number of capsules assessed in each category/the total number of retrieved capsules) × 100.

### Statistical analysis

Data are expressed as Mean ± SEM in these experiments. Paired Student’s *t*-test was used to compare two small sets of quantitative data from surface roughness, mechanical property, TLRs studies, protein adsorption, macrophage activation studies, and ex vivo GSIS experiments, with *P* < 0.05 being considered as statistically significant. For PCO quantification, Chi-squared test with Bonferroni correction was used to compare modified alginates to SLG20 alginate and calculate *P* values. Differences between groups were considered statistically significant when *P* < 0.05.

### Reporting summary

Further information on research design is available in the Nature Research Reporting Summary linked to this article.

## Supplementary information

Supplementary information

Supplementary Movie 1

Supplementary Movie 2

Reporting Summary

## Data Availability

All relevant data are available within the article and Supplementary Information, and from the corresponding author upon reasonable request.
