# Supplementary material for: Zwitterionically modified alginates mitigate cellular overgrowth for cell encapsulation
Source: Nat Commun. 2019 Nov 20;10:5262. doi: 10.1038/s41467-019-13238-7 (PMC6868136; doi:10.1038/s41467-019-13238-7)
Supplement: Supplementary file 1 — Supplementary information [file 41467_2019_13238_MOESM1_ESM.pdf]

Zwitterionically modified alginates mitigate cellular overgrowth for cell encapsulation

Liu et al.

Supplementary information

Zwitterionically modified alginates mitigate cellular overgrowth for cell encapsulation

Authors: Qingsheng Liu[1], Alan Chiu[1], Long-Hai Wang[1], Duo An[1], Monica Zhong[1], Alexandra M Smink[2], Bart J de Haan[2], Paul de Vos[2], Kevin Keane[3], Andreas Vegge[4], Esther Y. Chen[5], Wei Song[1], Wendy F. Liu[5], James Flanders[6], Claude Rescan[7], Lars Groth Grunnet[7], Xi Wang[1], Minglin Ma[1,*]

Affiliations:

[1] Department of Biological and Environmental Engineering, Cornell University, Ithaca, New York 14853, USA

[2] Department of Pathology and Medical Biology, University of Groningen and University Medical Center Groningen, Groningen, Netherlands

[3] Stem Cell Biology, Novo Nordisk A/S, 2760 Måløv, Denmark

[4] Diabetes Research, Novo Nordisk A/S, 2760 Måløv, Denmark

[5] Department of Biomedical Engineering, University of California Irvine, Irvine, CA 92697, USA

[6] Department of Clinical Sciences, Cornell University, Ithaca, New York 14853, USA

[7] Stem Cell Pharmacology, Novo Nordisk A/S, 2760 Måløv, Denmark

*Correspondence and requests for materials should be addressed to M.M. (email: mm826@cornell.edu

Supplementary Tables

	SLG20	SB-VLVG	SB-SLG20	SB-SLG100	CB1-SLG20	CB2-SLG20
14 days implantation	84.5 ± 3.7	83.9 ± 1.5	85.3 ± 3.0	89.3 ± 2.1	90.8 ± 2.1	88.6 ± 3.9
100 days implantation	80.1 ± 2.6	−	80.3 ± 5.7	86.5 ± 2.3	86.8 ± 4.0	90.7 ± 1.5
180 days implantation	77.8 ± 3.8	−	84.3 ± 3.7	−	−	−

Supplementary Table 1. The retrieval rate of empty capsules in C57BL/6J mice at various post-implantation time points.

The retrieval rate (%) was quantified using the following equation:

(The volume of retrieved capsules / The total volume of capsules before implantation) ×100

It should also be pointed out that there were two key factors affecting accuracy of the retrieval rate calculated by volumes. First, it was found that the size of capsules became slightly smaller after retrieval especially for long-term studies possibly due to change of environment from body fluid to a Ca^{2+}-containing stabilizing buffer that was used to collect the microcapsules. Second, protein adsorption and cellular deposition on the capsules tended to slightly increase the size. For zwitterionically modified alginate with little cellular deposition, the actual retrieval rate should be a little higher than our calculated value. For unmodified alginate, the two factors mentioned above co-existed and therefore the actual retrieval rate was estimated to be more or less the same as the calculated value.

From Supplementary Table 1, we can see that the retrieval rate for modified microcapsules was in general comparable to or higher than the unmodified microcapsules. The SB-VLVG microcapsules had slightly lower retrieval rate because some of the capsules were broken or partially dissolved due to the lowest molecular weight of VLVG among all alginates.

	SLG20	SB-SLG20
90 days implantation	74.3 ± 5.7	82.3 ± 5.5
200 days implantation	66.3 ± 6.5	83.3 ± 5.8

Supplementary Table 2. The retrieval rate of islet-containing capsules in C57BL/6J mice at various post-implantation time points.

The retrieval rate (%) was quantified using the following equation:

(The volume of retrieved capsules / The total volume of capsules before implantation) ×100

The retrieval rate for the microcapsules containing islets was significantly higher for the modified group than the unmodified (Supplementary Table 2), consistent with the better biocompatibility and diabetes correction.

Name of the cell line (InvivoGen)	Agonist (InvivoGen)	Selection antibiotics (InvivoGen)
HEK-Blue^{TM} hTLR4	10 ng/ml LPS	HEK-Blue^{TM} Selection (1X)
HEK-Blue^{TM} hTLR2	10^7 ml heat-killed Listeria monocytogenes (HKLM)	HEK-Blue^{TM} Selection (1X)

Supplementary Table 3. The TLR reporter cell lines, their agonists and selection antibiotics.

Supplementary Figures

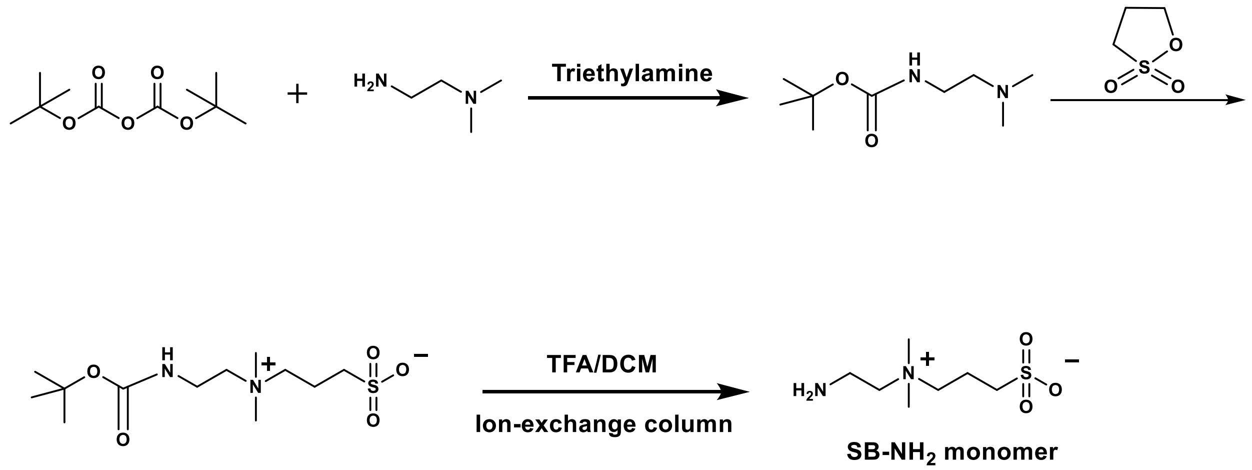

Supplementary Figure 1. Synthetic pathway of SB-NH_2 monomer.

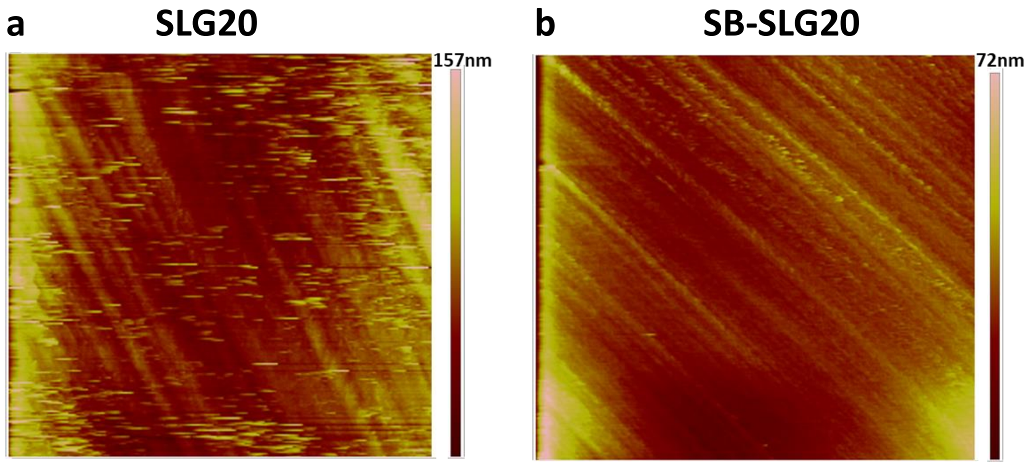

Supplementary Figure 2. Representative AFM images of (a) SLG20 and (b) SB-SLG20 alginate microcapsules (n=3 per group).

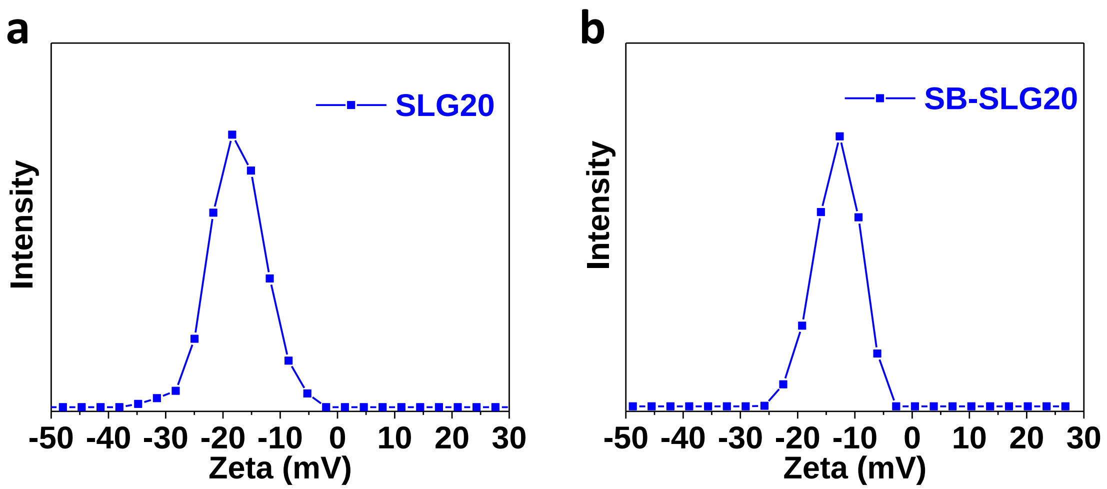

Supplementary Figure 3. Zeta potentials of (a) SLG20 and (b) SB-SLG20 alginate nanogels (n=5 per group).

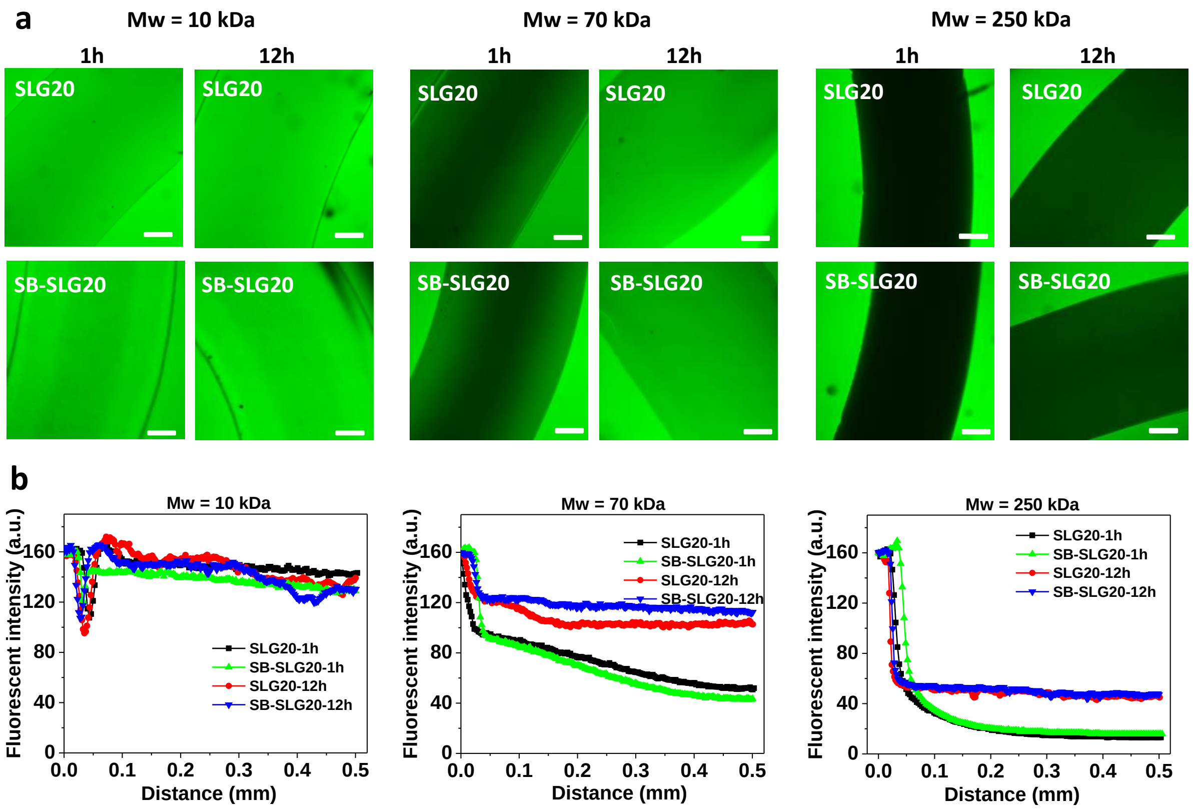

Supplementary Figure 4. The diffusion measurement of SLG20 and SB-SLG20 alginate hydrogels (n=3 per group). Representative fluorescent images (a) and intensity profiles (b) showing the permeability of alginate hydrogel to different molecular weight FITC-dextrans. Scale bars: 200 µm.

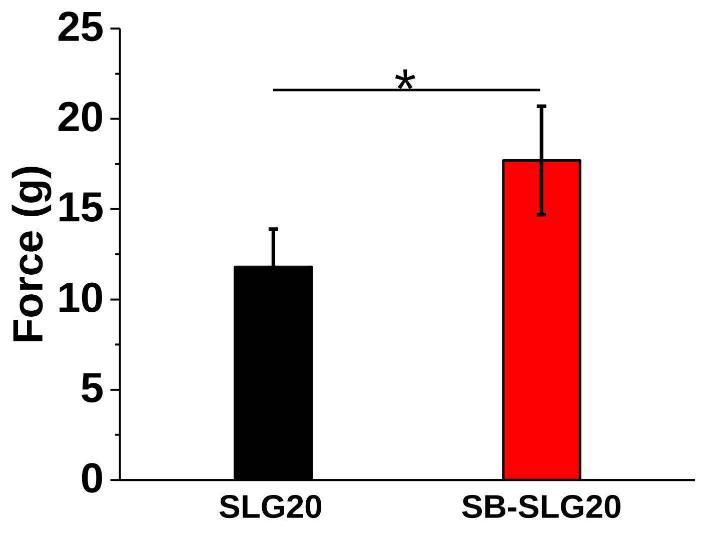

Supplementary Figure 5. Mechanical property of alginate microcapsules (n=10 per group). The data are Mean ± SEM, and error bars indicate the SEM. Paired Student's t-test is used to compare two small sets of quantitative data. *P < 0.05.

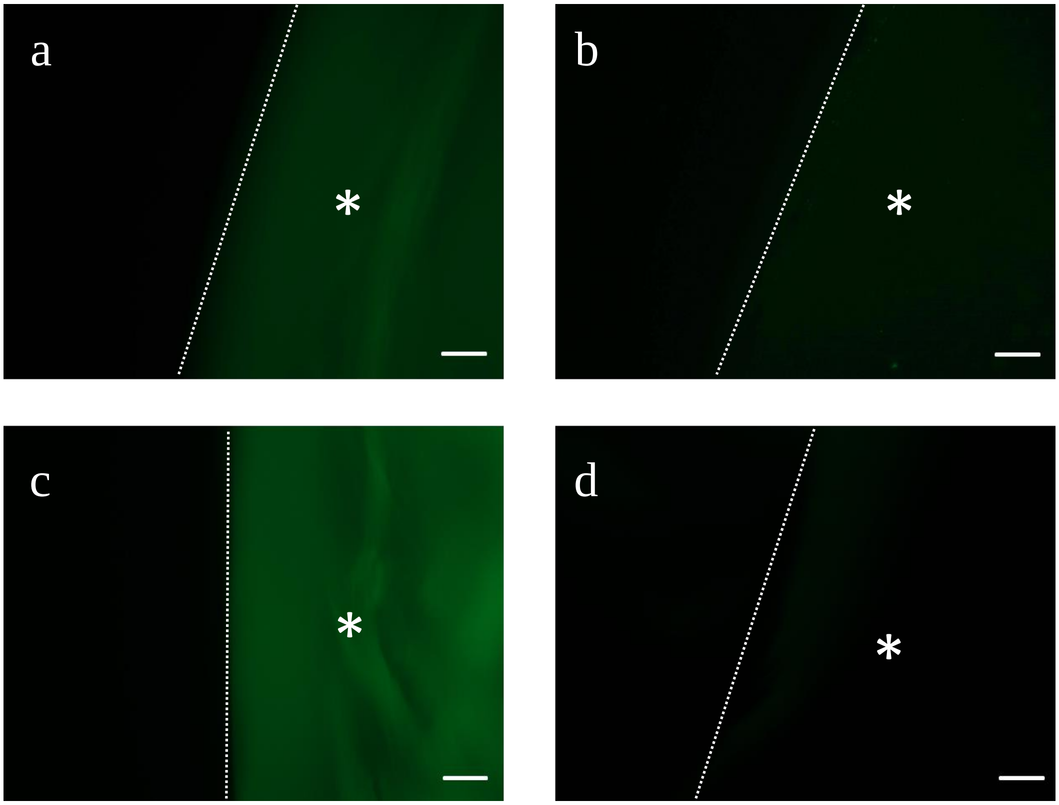

Supplementary Figure 6. Fluorescence microscope images of FITC-labelled fibrinogen adsorption on the (a) SLG20 hydrogels and (b) SB-SLG20 hydrogels, and FITC-labelled lysozyme adsorption on the (c) SLG20 hydrogels and (d) SB-SLG20 hydrogels. Scale bars: 100 µm; asterisks indicate the location of the alginate hydrogels and dashed lines indicate the edge of alginate hydrogels.

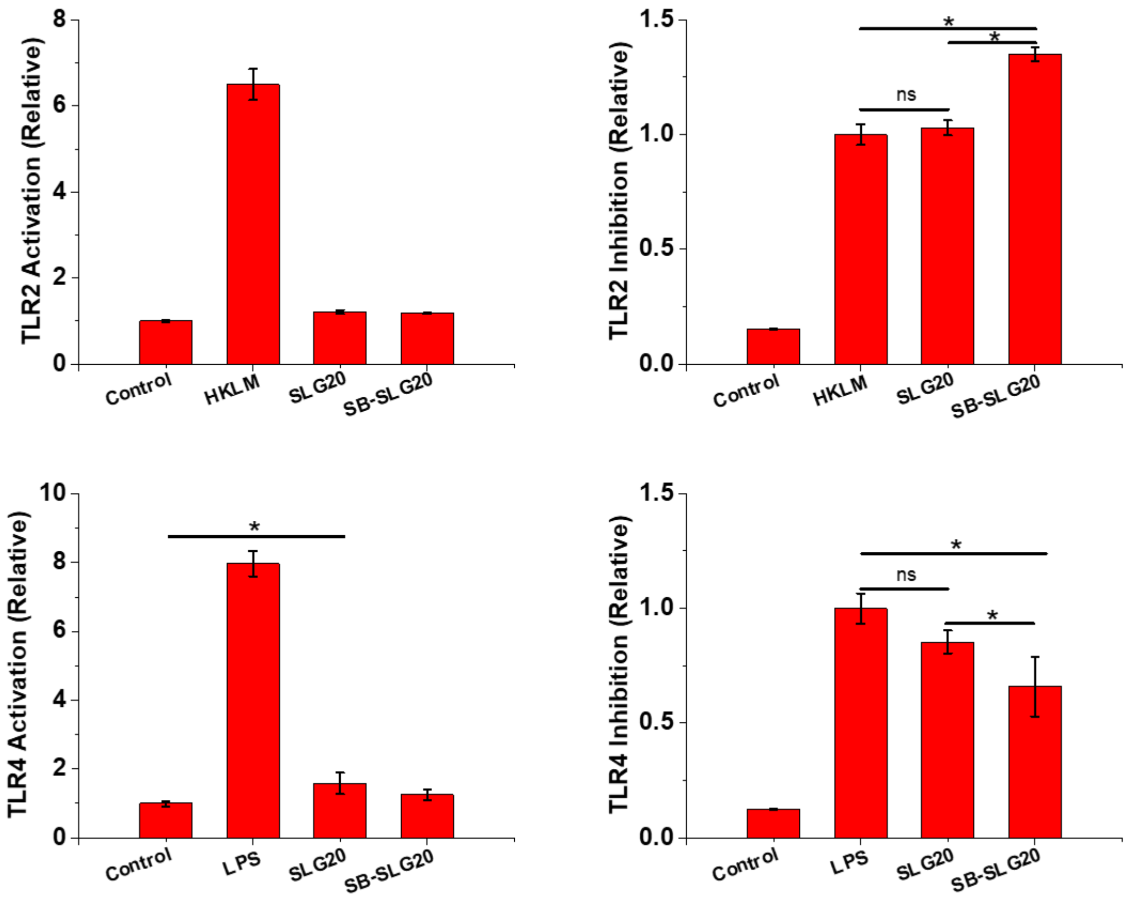

Supplementary Figure 7. Immune TLR2 and TLR4 activation and inhibition by SLG20 and SB-SLG20 hydrogel capsules. Both cell lines were incubated with alginate capsules, culture medium as negative control, and Lipopolysaccharides (LPS) or heat killed *Listeria monocytogenes* (HKLM) as positive controls respectively. n = 6 per group. The data are Mean ± SEM, and error bars indicate the SEM. Paired Student's t-test is used to compare two small sets of quantitative data. *$P < 0.05$; ns, not significant.

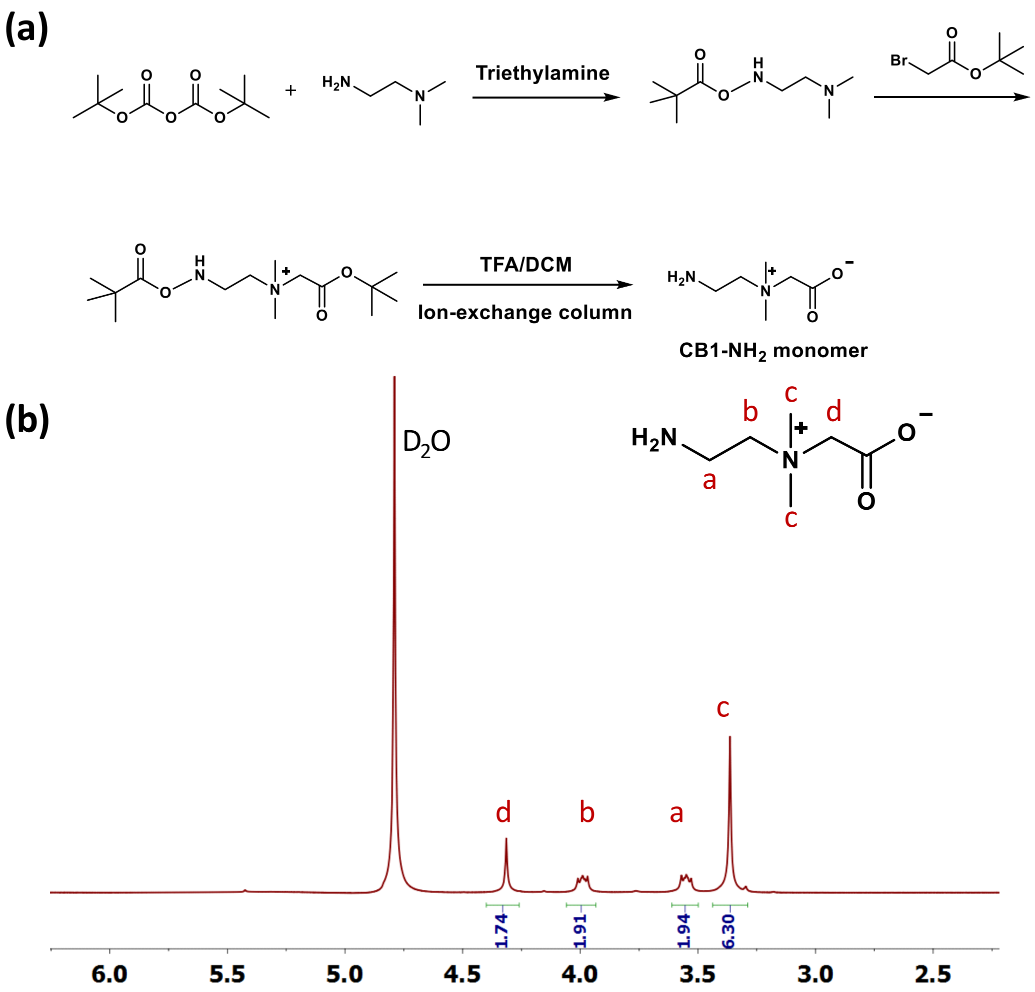

Supplementary Figure 8. Synthetic pathway (a) and ^{1}H NMR (b) characterization of CB1-NH_2 monomer.

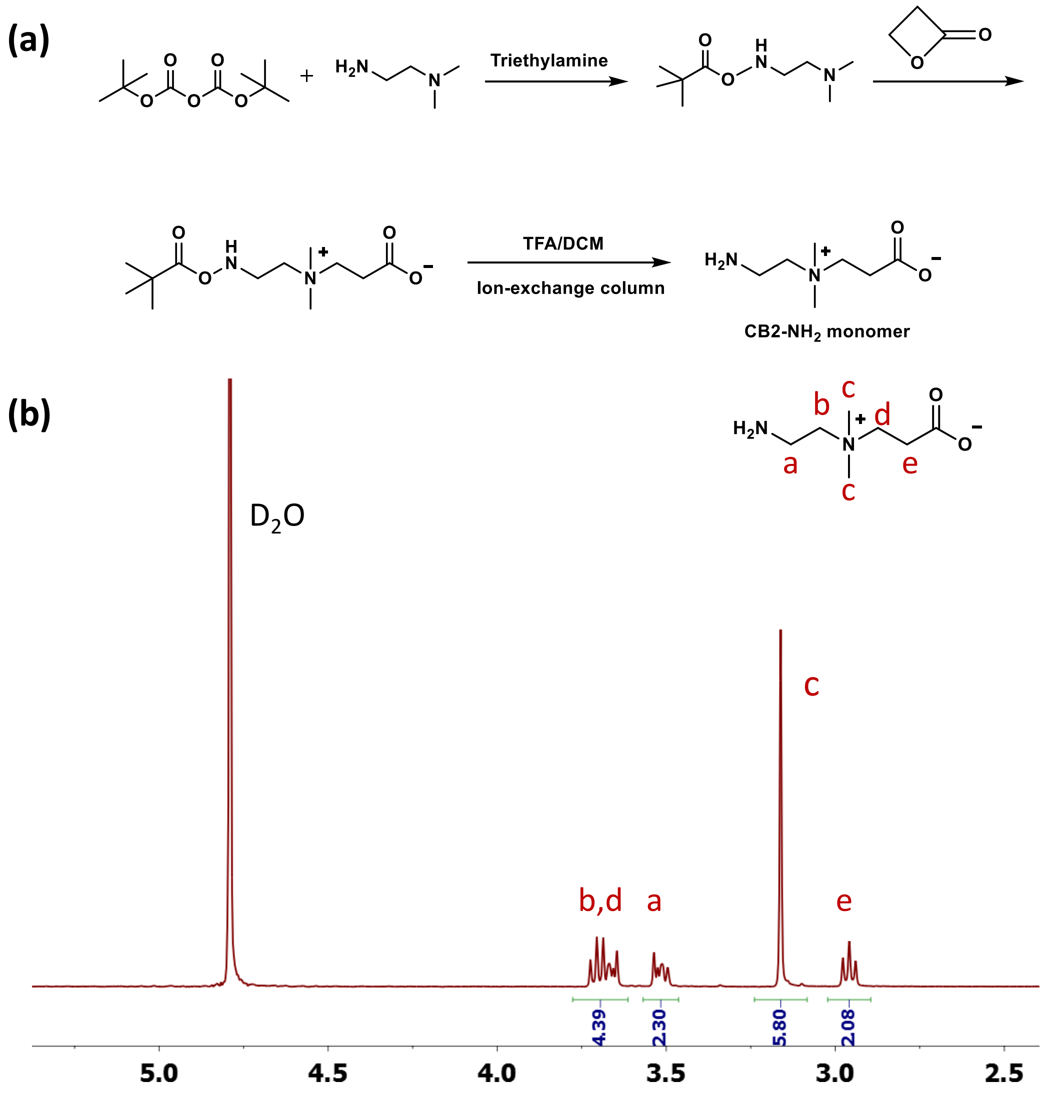

Supplementary Figure 9. Synthetic pathway (a) and ^{1}H NMR (b) characterization of CB2-NH_2 monomer.

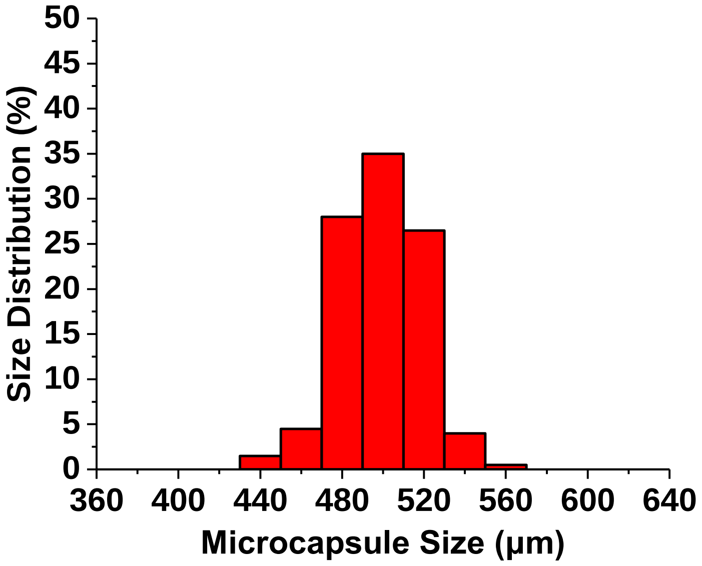

Supplementary Figure 10. Size distribution of SB-modified alginate capsules for mouse studies.

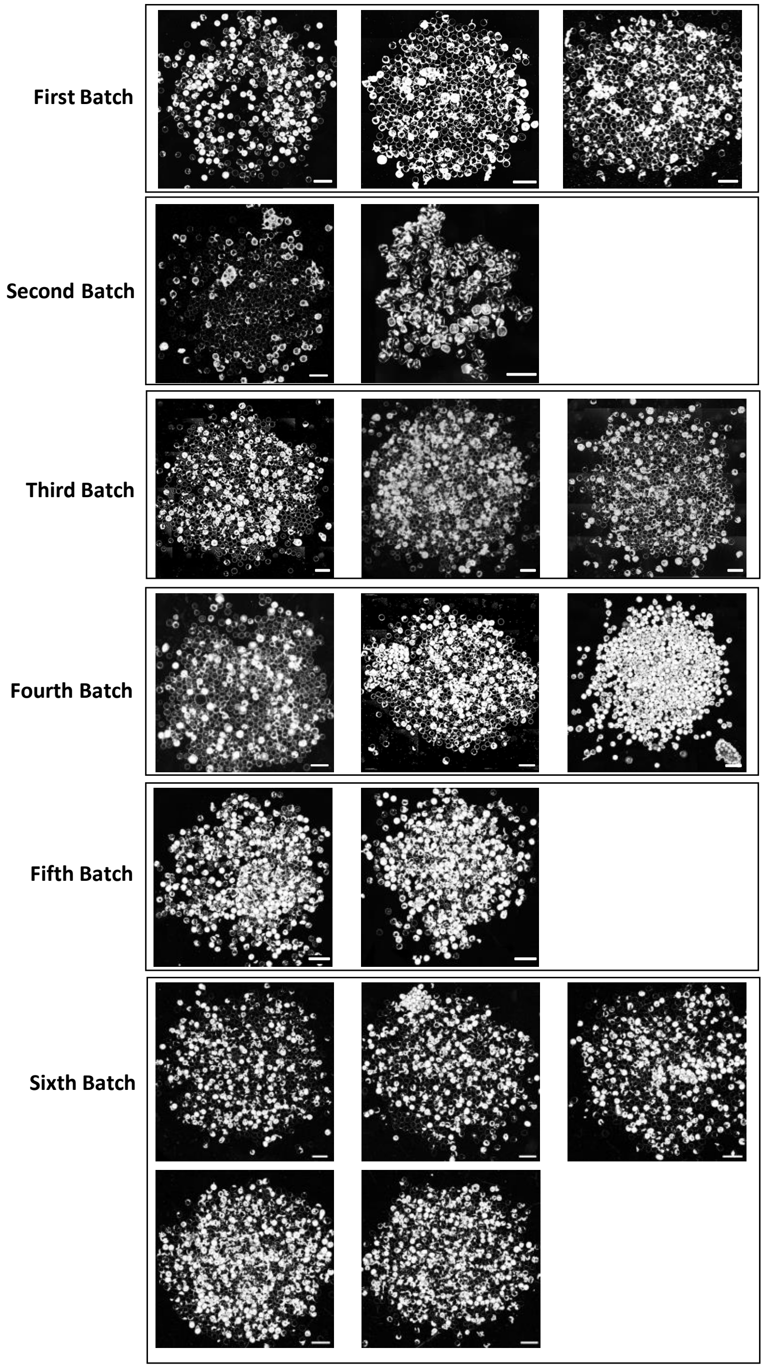

Supplementary Figure 11. Phase contrast images of retrieved SLG20 microcapsules, 14 d post intraperitoneal implantation in C57BL/6J mice (Scale bars, 2 mm). Each image represents all microcapsules retrieved from 1 mouse, and each panel represents one batch of experiment. (It is noted that the first batch was performed in the experiment presented in Supplementary Figure 13 and the images are also shown there for comparison.)

First Batch

Second Batch

Third Batch

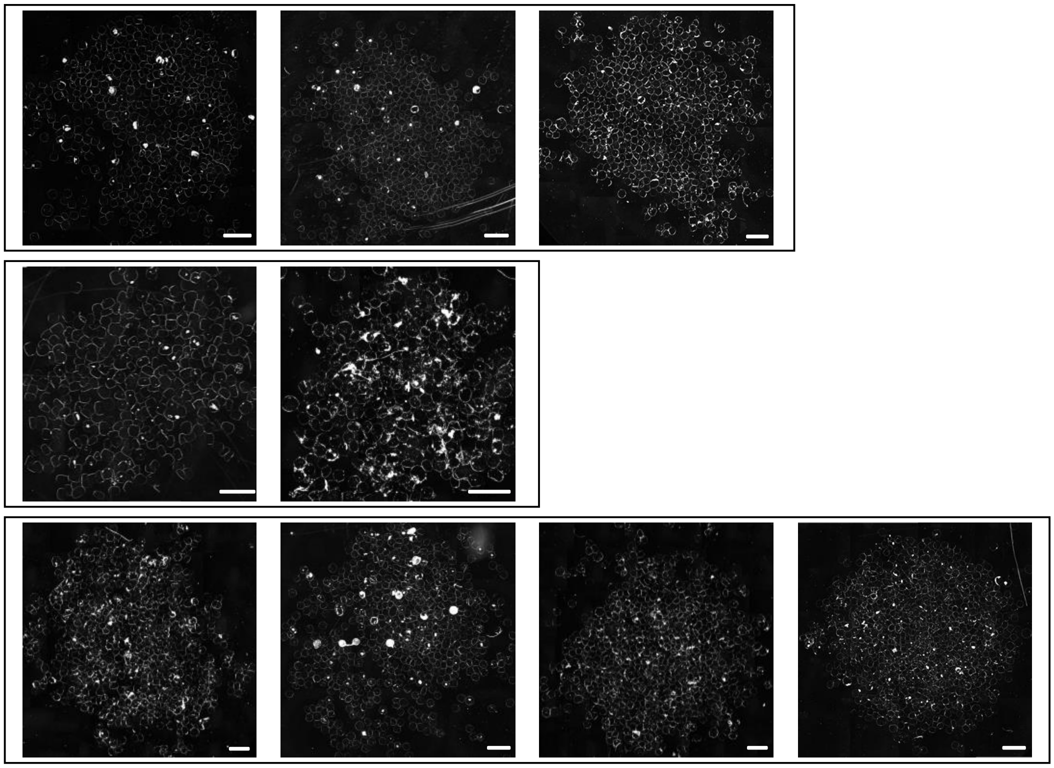

Supplementary Figure 12. Phase contrast images of retrieved SB-VLVG microcapsules, 14 d post intraperitoneal implantation in C57BL/6J mice (Scale bars, 2 mm). Each image represents all microcapsules retrieved from 1 mouse, and each panel represents one batch of experiment. (It is noted that the first batch was performed in the experiment presented in Supplementary Figure 13 and the images are also shown there for comparison.)

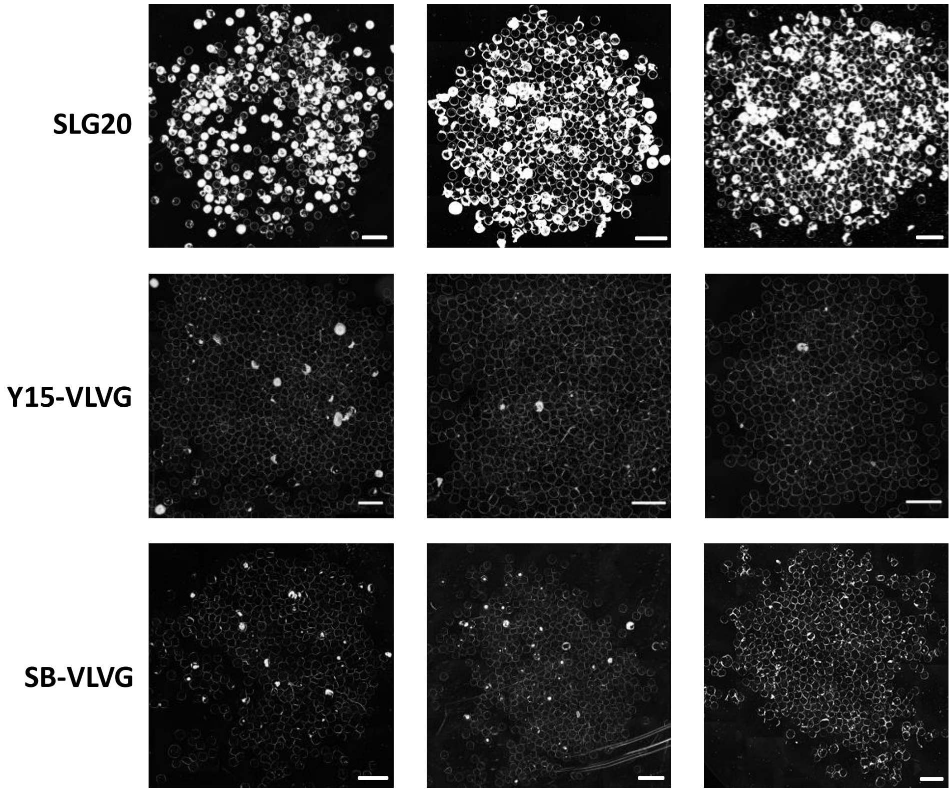

Supplementary Figure 13. Phase contrast images of various retrieved alginate hydrogels, 14 d post intraperitoneal implantation in C57BL/6J mice (Scale bars, 2 mm). Each image represents all microcapsules retrieved from 1 mouse. (It is noted that the phase-contrast images of SLG20 and SB-VLVG microcapsules are also presented in Supplementary Figure 11 and 12, respectively.)

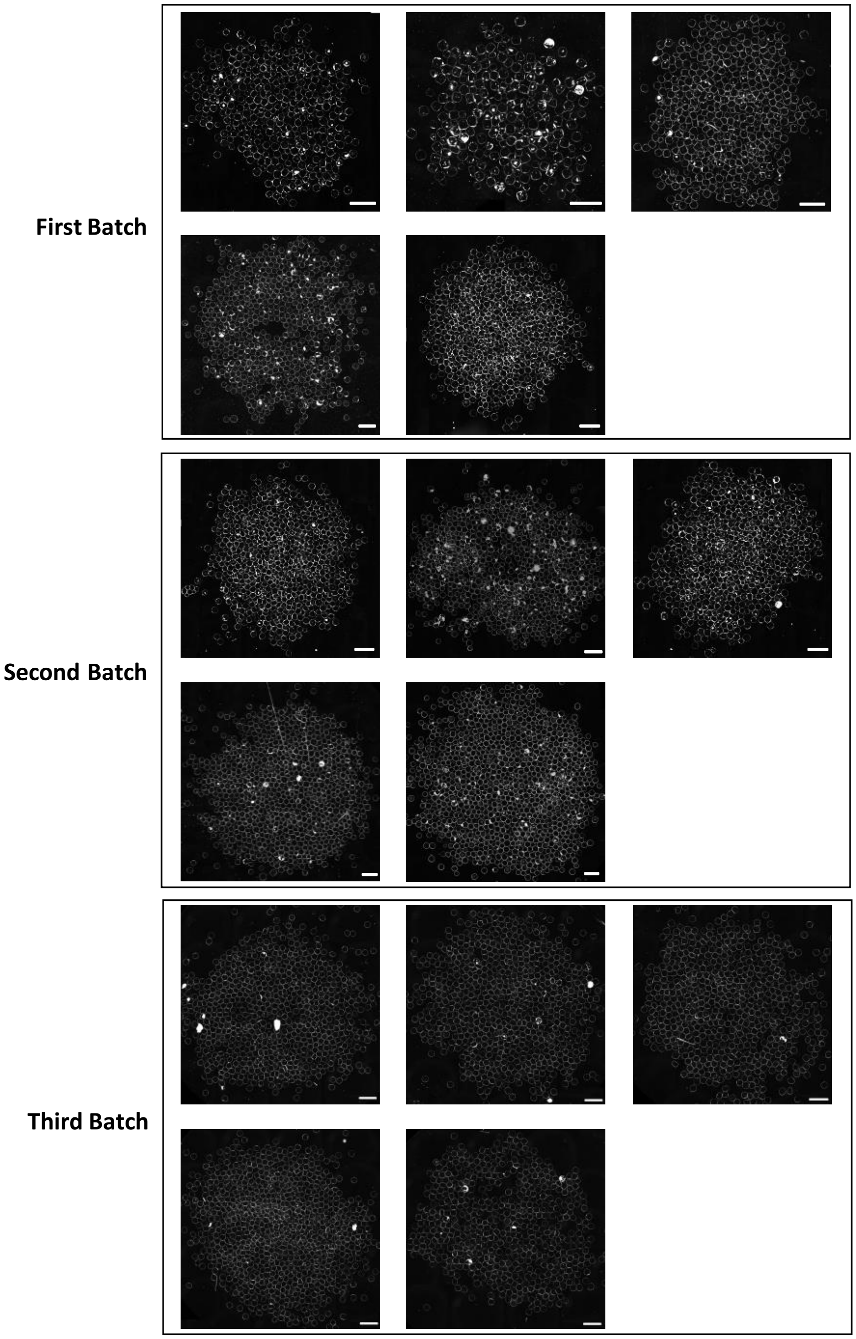

Supplementary Figure 14. Phase contrast images of retrieved SB-SLG20 microcapsules, 14 d post intraperitoneal implantation in C57BL/6J mice (Scale bars, 2 mm). Each image represents all microcapsules retrieved from 1 mouse, and each panel represents one batch of experiment.

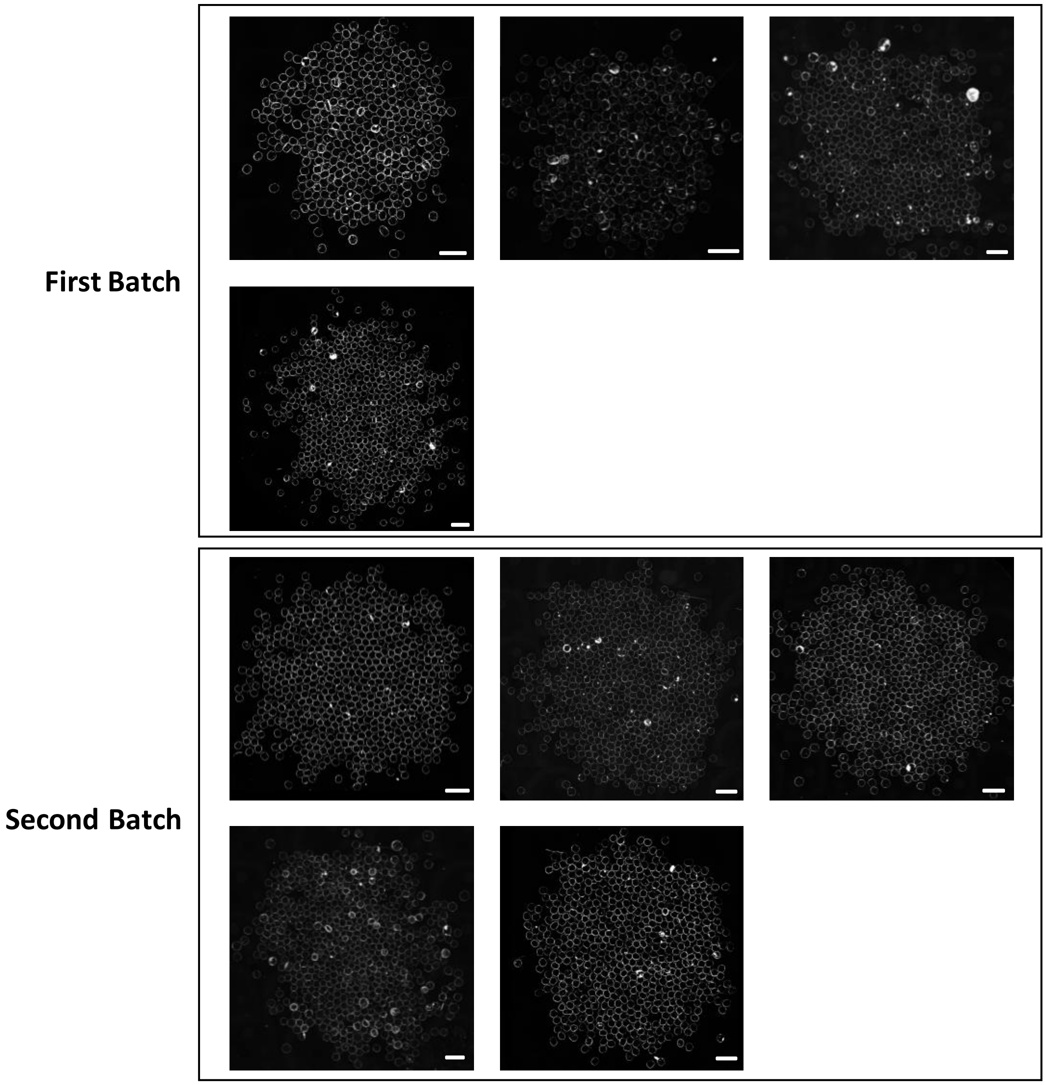

Supplementary Figure 15. Phase contrast images of retrieved SB-SLG100 microcapsules, 14 d post intraperitoneal implantation in C57BL/6J mice (Scale bars, 2 mm). Each image represents all microcapsules retrieved from 1 mouse, and each panel represents one batch of experiment.

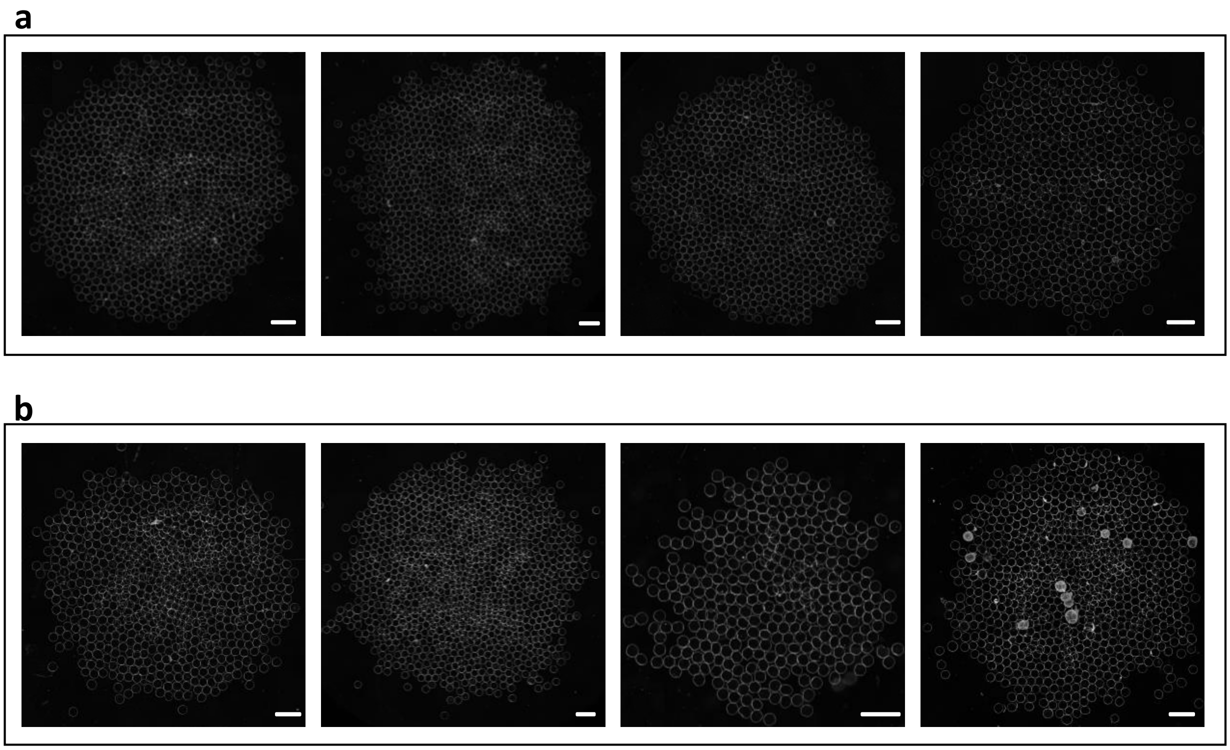

Supplementary Figure 16. Phase contrast images of retrieved (a) CB1-SLG20 and (b) CB2-SLG20 microcapsules, 14 d post intraperitoneal implantation in C57BL/6J mice (Scale bars, 2 mm). Each image represents all microcapsules retrieved from 1 mouse, and each panel represents one batch of experiment.

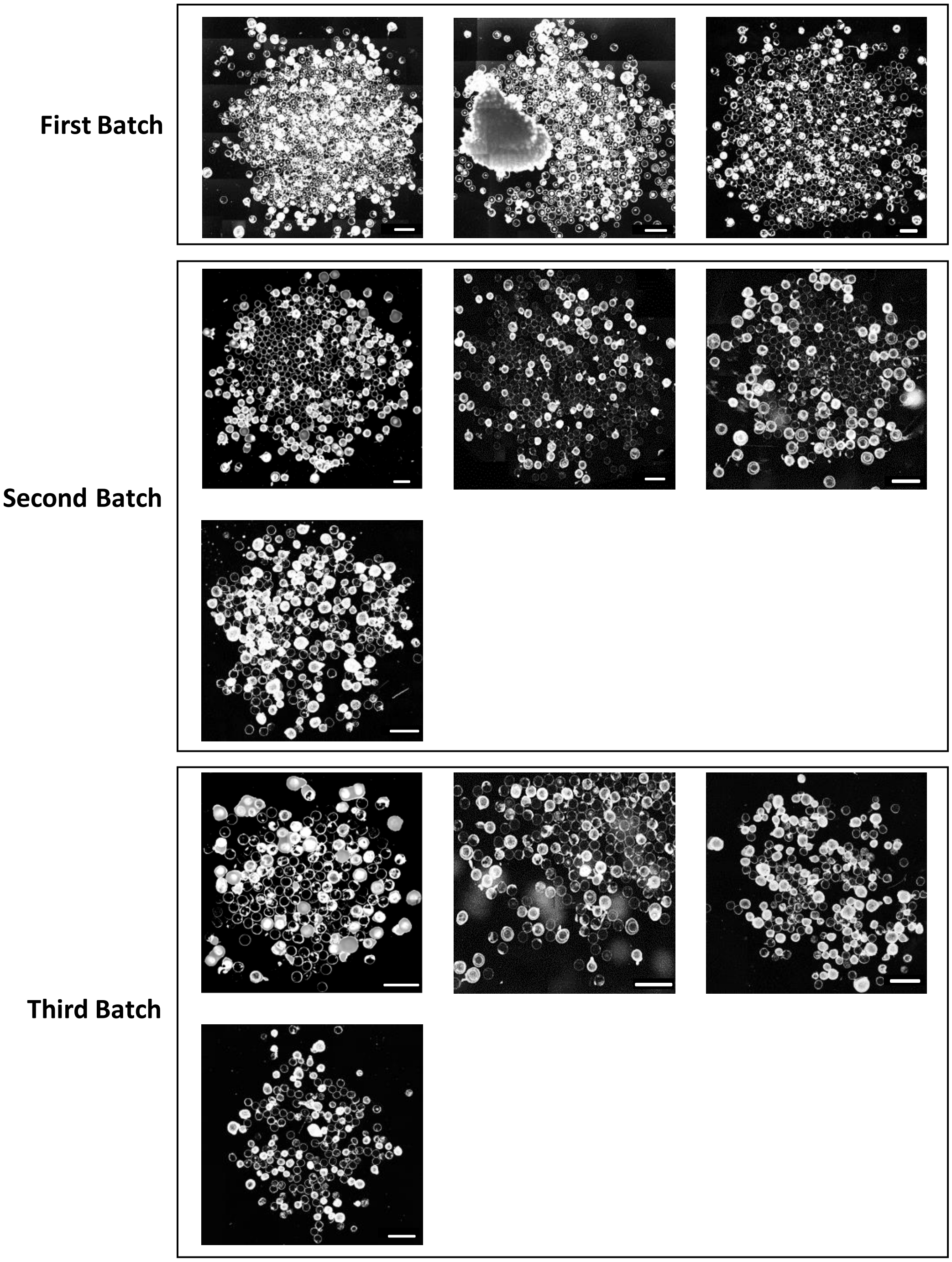

Supplementary Figure 17. Phase contrast images of retrieved SLG20 microcapsules, 100 d post intraperitoneal implantation in C57BL/6J mice (Scale bars, 2 mm). Each image represents all microcapsules retrieved from 1 mouse, and each panel represents one batch of experiment.

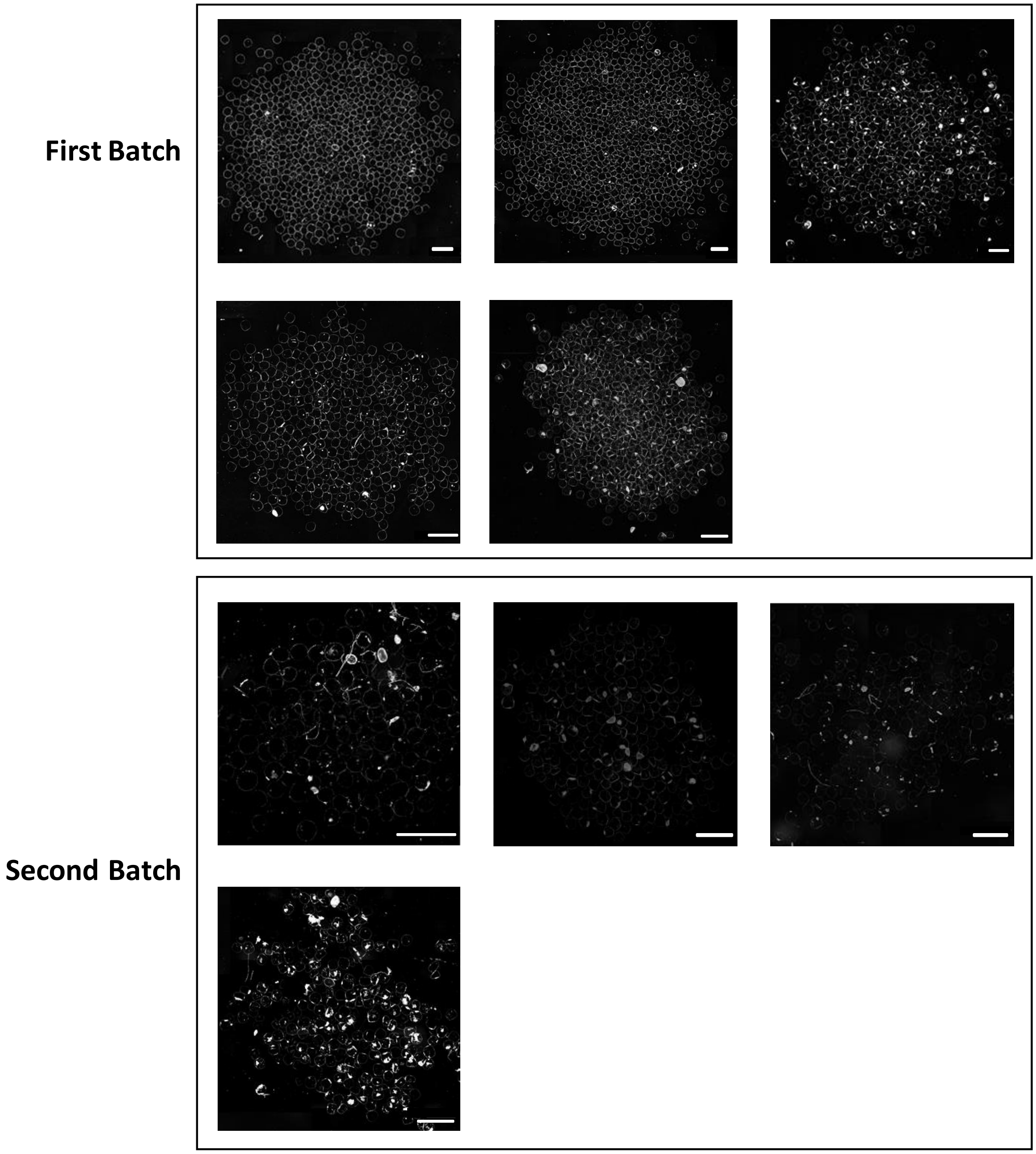

Supplementary Figure 18. Phase contrast images of retrieved SB-SLG20 microcapsules, 100 d post intraperitoneal implantation in C57BL/6J mice (Scale bars, 2 mm). Each image represents all microcapsules retrieved from 1 mouse, and each panel represents one batch of experiment.

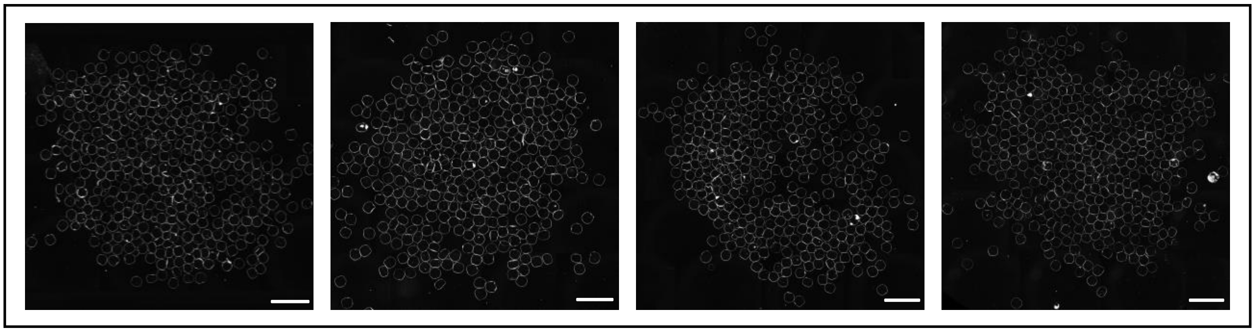

Supplementary Figure 19. Phase contrast images of retrieved SB-SLG100 microcapsules, 100 d post intraperitoneal implantation in C57BL/6J mice (Scale bars, 2 mm). Each image represents all microcapsules retrieved from 1 mouse.

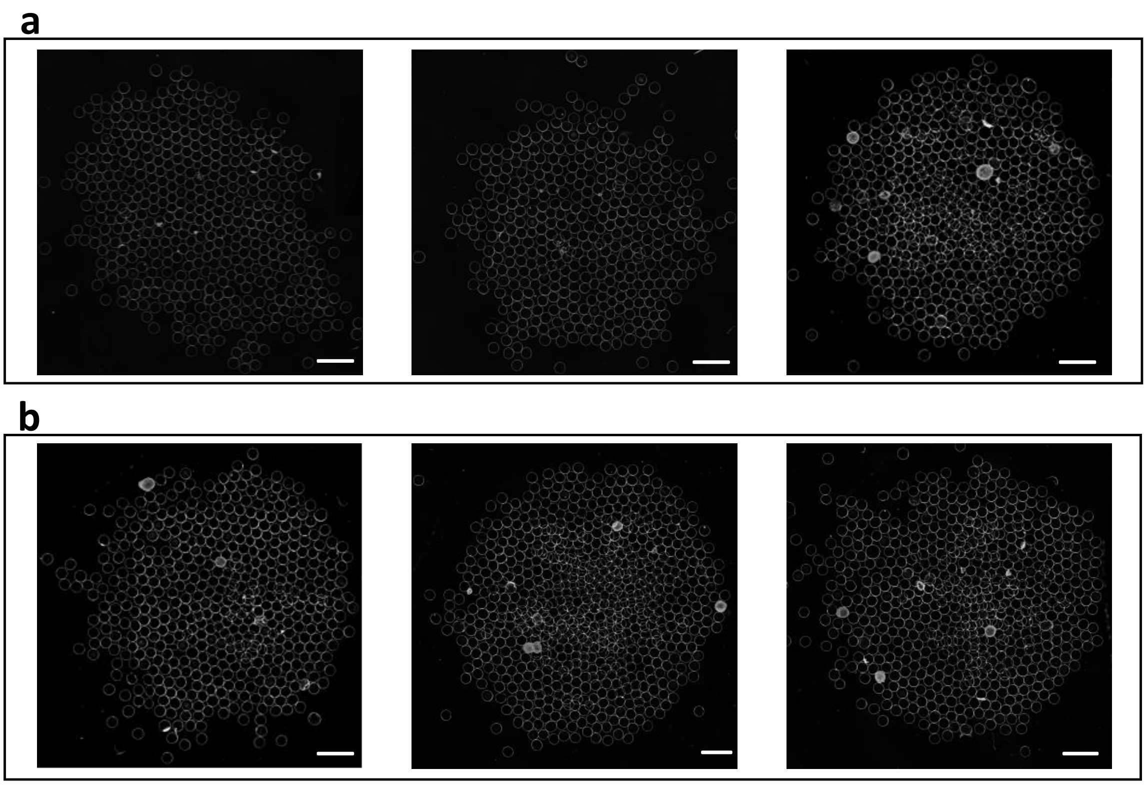

Supplementary Figure 20. Phase contrast images of retrieved (a) CB1-SLG20 and (b) CB2-SLG20 microcapsules, 100 d post intraperitoneal implantation in C57BL/6J mice (Scale bars, 2 mm). Each image represents all microcapsules retrieved from 1 mouse.

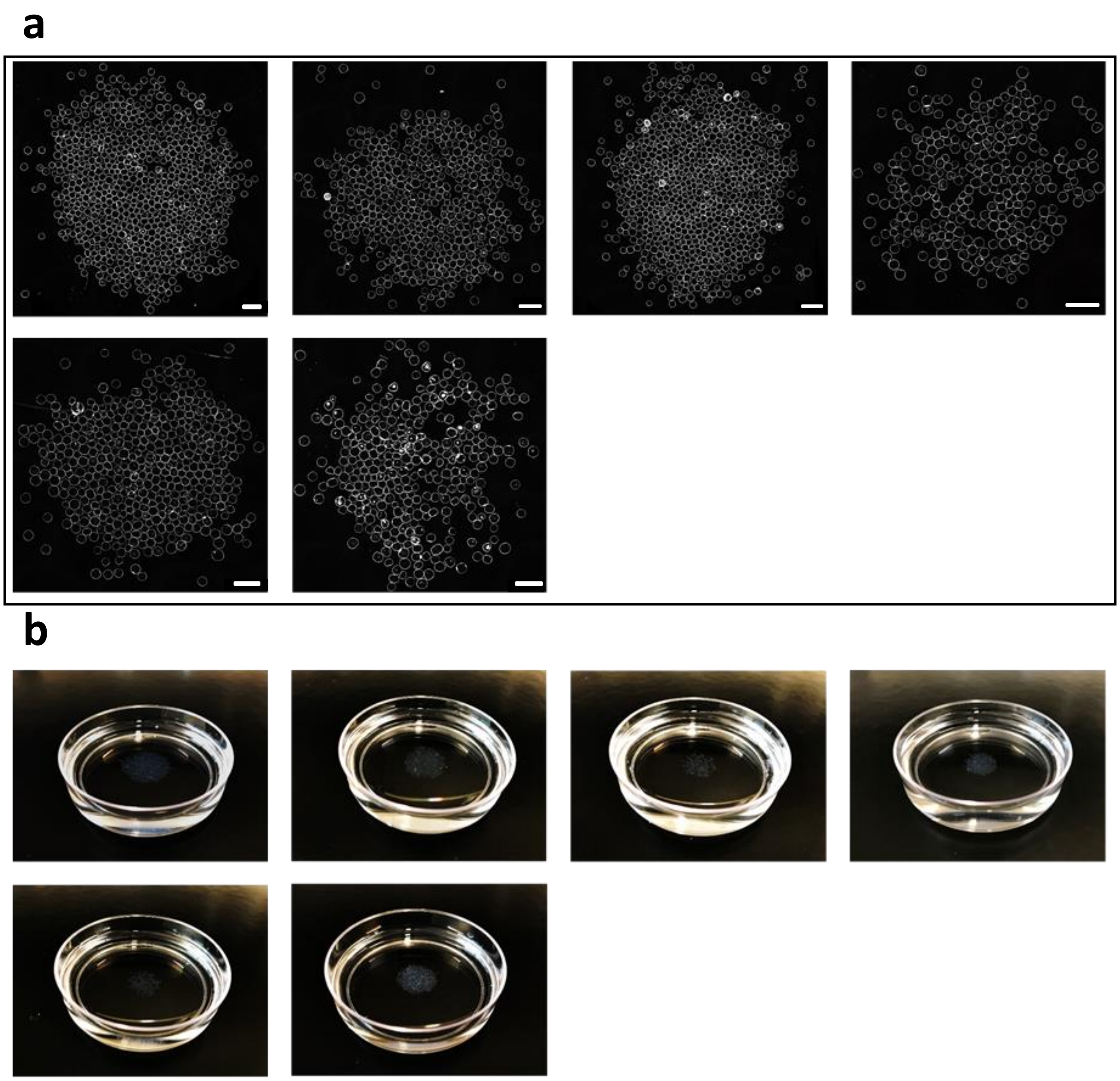

Supplementary Figure 21. (a) Phase contrast images of retrieved SB-SLG20 microcapsules, 180 d post intraperitoneal implantation in C57BL/6J mice (Scale bars, 2 mm). (b) Retrieved SB-SLG20 microcapsules in a Petri dish. Each image represents all microcapsules retrieved from 1 mouse.

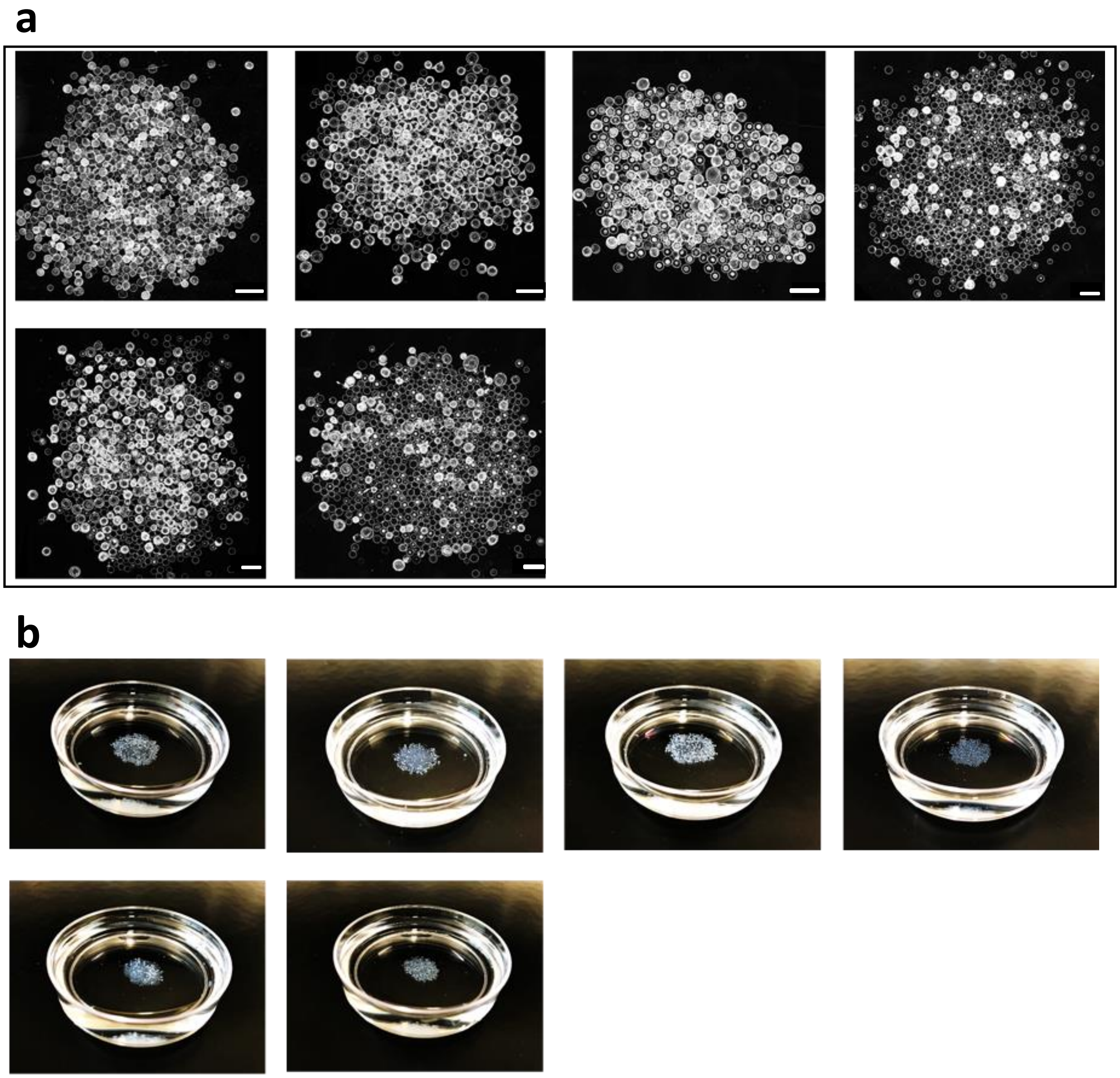

Supplementary Figure 22. (a) Phase contrast images of retrieved SLG20 microcapsules, 180 d post intraperitoneal implantation in C57BL/6J mice (Scale bars, 2 mm). (b) Retrieved SLG20 microcapsules in a Petri dish. Each image represents all microcapsules retrieved from 1 mouse.

Supplementary Discussion

To investigate what kind of cells attached to the microcapsules, the retrieved microcapsules after 2 weeks of implantation were then stained by a number of cellular markers including α-smooth muscle actin (SMA), CD68, F4/80, CD11b and Ly-6G/Ly-6C. It is noted that only a small fraction of the SB-SLG20 capsules had any cell attachment that were stainable for this characterization. For example, 2 out of 50 capsules for CD11b/CD68 Staining had cell attachment, and 2 out of 150 capsules for CD11b/Ly-6G Ly-6C Staining had cell attachment.

The retrieved microcapsules were co-stained with SMA (red) and CD68 (green) to demonstrate the distribution of the fibroblasts and macrophages (Supplementary Figure 23). Retrieved SLG20 microcapsules showed significantly more fibroblast and macrophage adhesion compared to zwitterion-modified microcapsules. The retrieved microcapsules were also co-stained with SMA (red) and F4/80 (green) to demonstrate the distribution of the fibroblasts and mature macrophages (Supplementary Figure 24). Retrieved SB-SLG20 microcapsules showed a significant reduction of fibroblast and macrophage adhesion compared to SLG20 microcapsules. Microcapsules were then co-stained with CD11b (red) and CD68 (green) to demonstrate the distribution of the monocytes, granulocytes, and macrophages (Supplementary Figure 25). CD11b stained for monocytes and granulocytes; CD68 stained for monocytes and macrophages. $CD11b^{+}CD68^{+}$(co-stained) cells are monocytes. $CD11b^{+}CD68^{-}$ (red only) cells are granulocytes. $CD11b^{-}CD68^{+}$ (green only) are macrophages and giant cells. The results indicated that retrieved SLG20 microcapsules had significantly more monocyte, granulocyte, and macrophage adhesion compared to SB-SLG20 microcapsules. To understand the granulocytes stained with CD11b, we co-stained microcapsule with CD11b and Ly-6G/Ly-6C (Gr-1) (Supplementary Figure 26). $CD11b^{+}$ Ly-6G/Ly-$6C^{high}$ (arrows) cells are neutrophils [1,2]. $CD11b^{+}$ Ly-6G/Ly-$6C^{low}$ and $CD11b^{+}$ Ly-6G/Ly-$6C^{-}$ cells are monocytes and macrophages.

In summary, these staining experiments (Supplementary Figure 23-26) revealed that the cells attached to microcapsules included monocytes, granulocytes, macrophages and fibroblasts, and there was a significant reduction of adhesion of these cells, particularly monocytes and neutrophils, after the zwitterionic modification consistent with the phase-contract images.

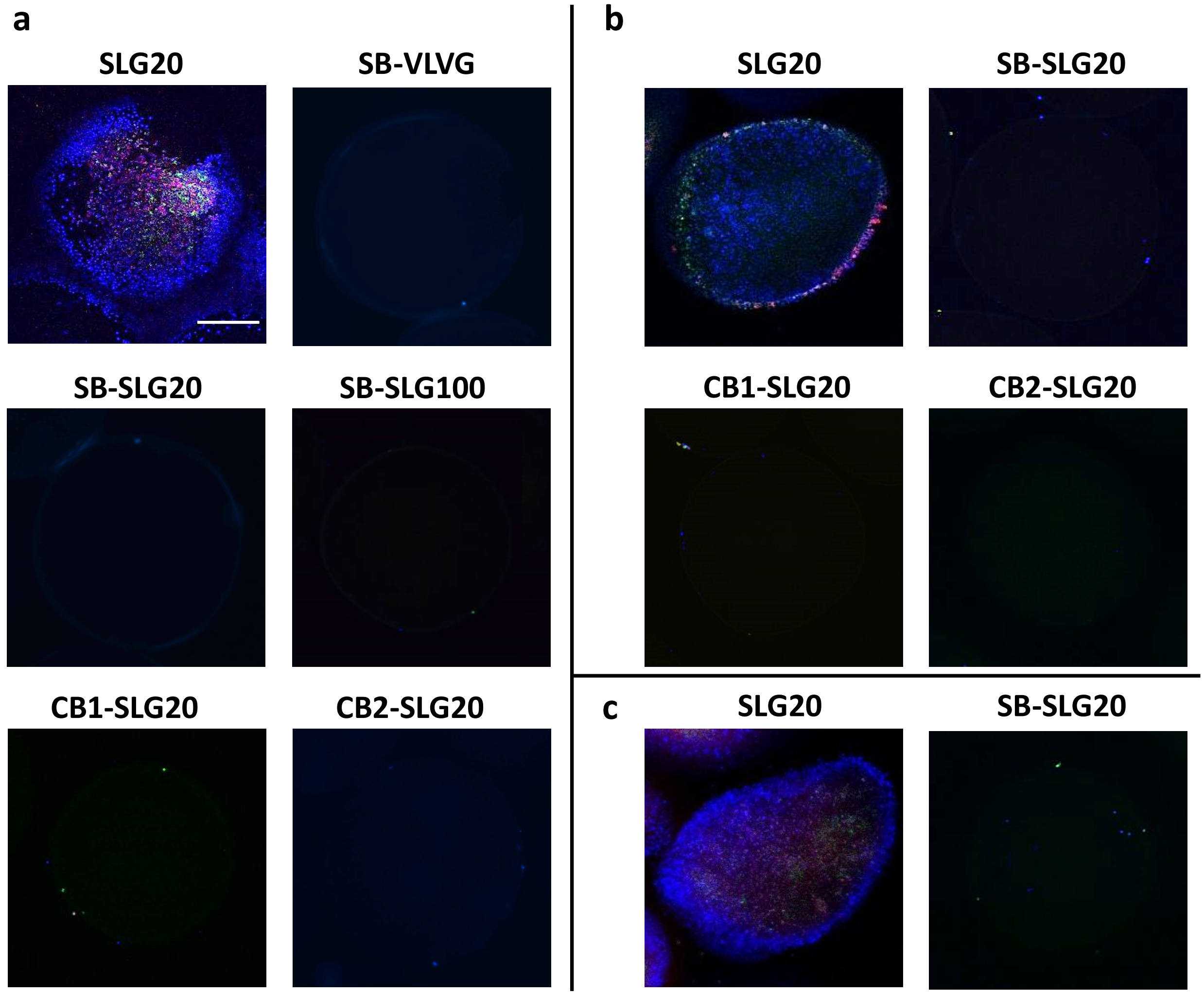

Supplementary Figure 23. Immunofluorescence confocal images of retrieved alginate microcapsules, (a) 14 d, (b) 100 d and (c) 180 d post intraperitoneal implantation in C57BL/6J mice. All the microcapsules were stained for macrophage markers (CD68, green), myofibroblast markers (α-smooth muscle actin, SMA, red) and general cellular deposition (DAPI, blue). Scale bar, 200 μm.

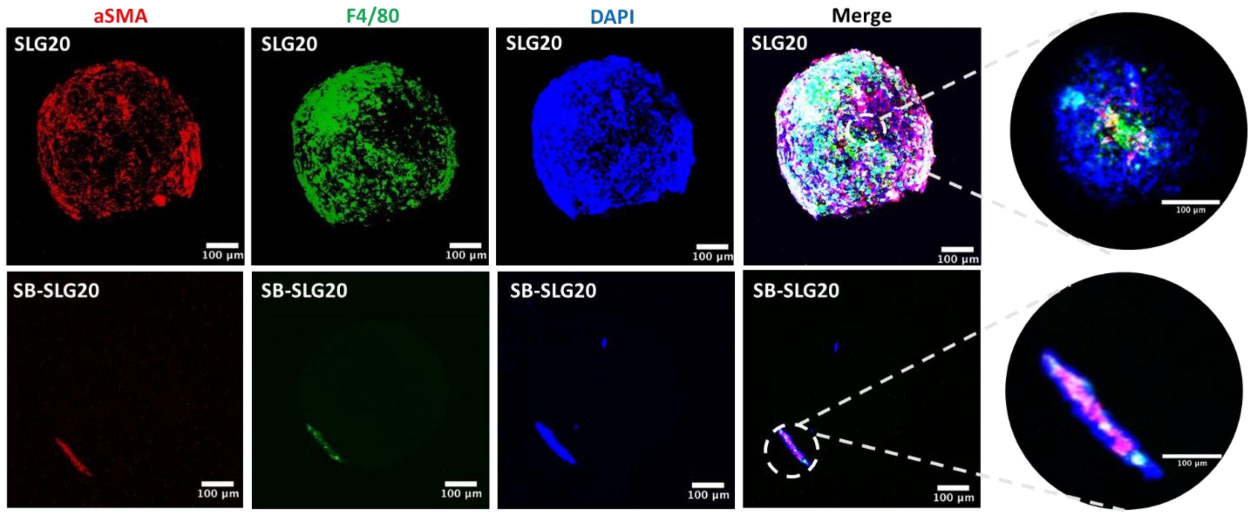

Supplementary Figure 24. Immunofluorescence confocal images of retrieved alginate microcapsules, 14 d post intraperitoneal implantation in C57BL/6J mice. All the microcapsules were stained for myofibroblast markers (α-smooth muscle actin, SMA, red), macrophage markers (F4/80, green), and general cellular deposition (DAPI, blue).

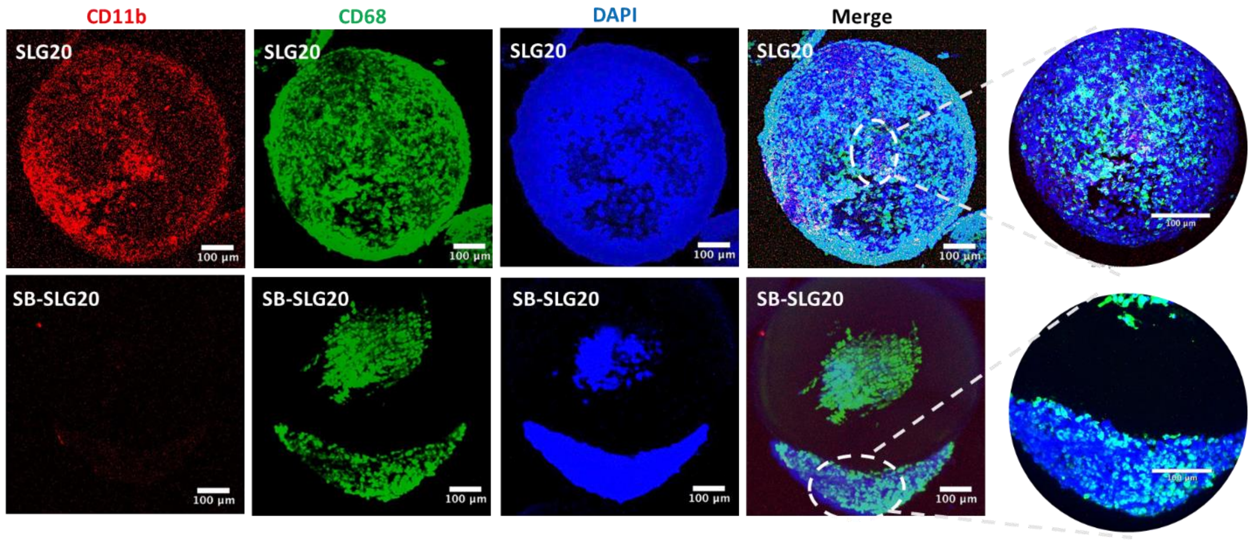

Supplementary Figure 25. Immunofluorescence confocal images of retrieved alginate microcapsules, 14 d post intraperitoneal implantation in C57BL/6J mice. All the microcapsules were stained for monocytes and granulocytes (CD11b, red), monocytes and macrophages (CD68, green), and general cellular deposition (DAPI, blue).

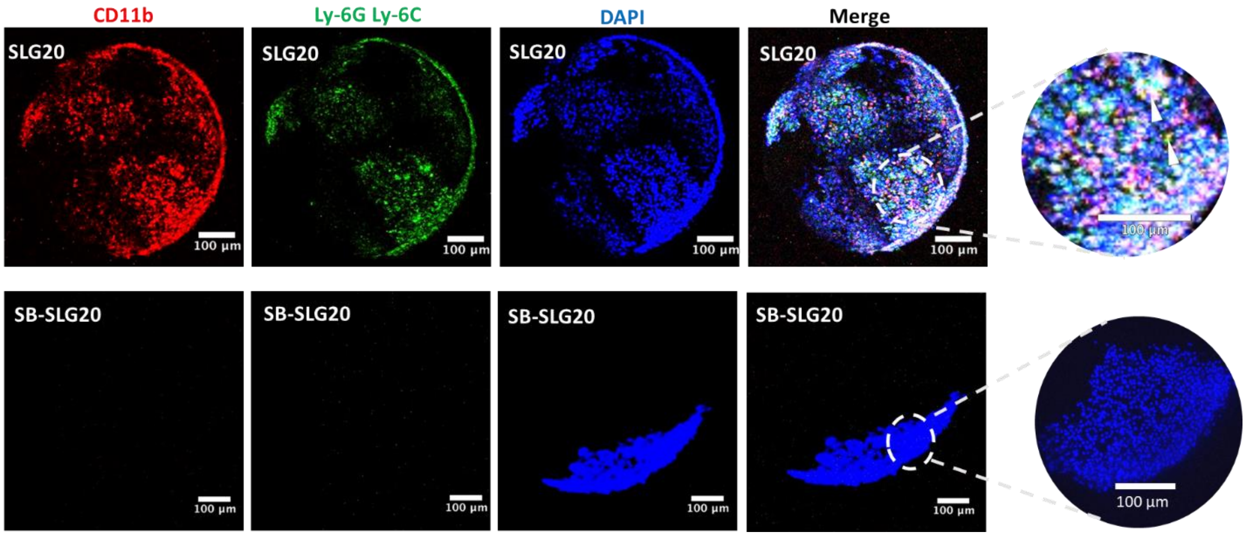

Supplementary Figure 26. Immunofluorescence confocal images of retrieved alginate microcapsules, 14 d post intraperitoneal implantation in C57BL/6J mice. All the microcapsules were stained for CD11b (red), Ly-6G/Ly-6C (green), and general cellular deposition (DAPI, blue). The arrows are neutrophils.

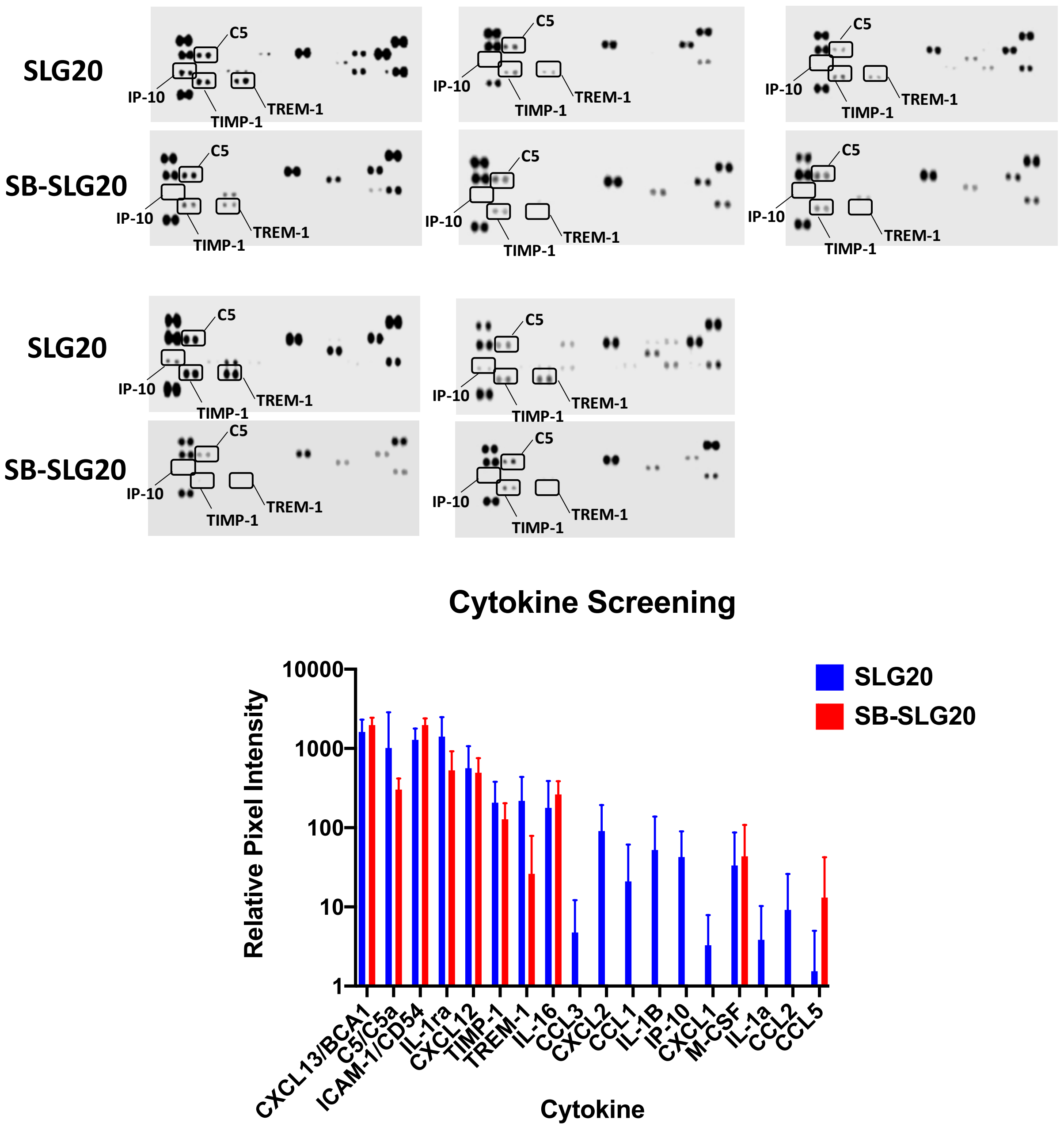

Supplementary Figure 27. Cytokine profiling for intraperitoneal liquid, 14 d post intraperitoneal implantation in C57BL/6J mice (n = 5 per group). The data are Mean ± SEM, and error bars indicate the SEM.

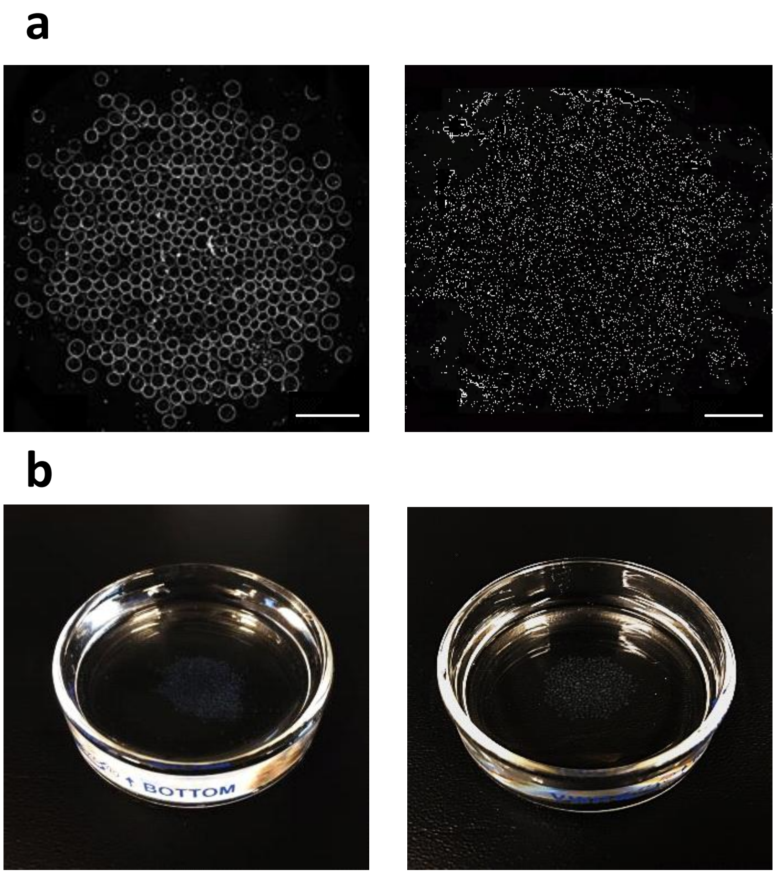

Supplementary Figure 28. (a) Phase contrast images of retrieved SB-SLG20 alginate microcapsules, 45 d post intraperitoneal implantation in dogs (Scale bars, 2 mm). (b) Retrieved SB-SLG20 microcapsules in a Petri dish. Each image represents microcapsules retrieved from 1 dog.

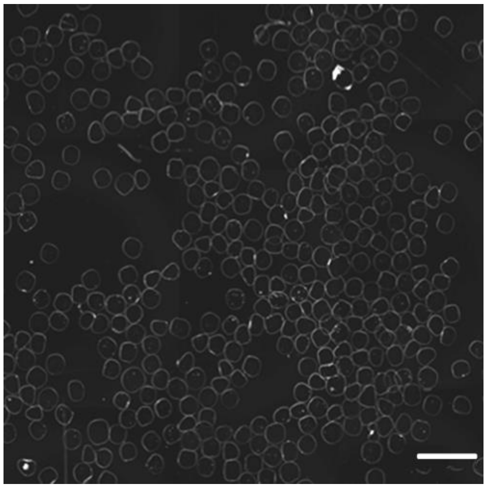

Supplementary Figure 29. Phase contrast images of retrieved SB-SLG20 alginate microcapsules, 90 d post intraperitoneal implantation in dogs (Scale bars, 2 mm). Each of the images (Figure 4i and Supplementary Figure 29) represents microcapsules retrieved from 1 dog.

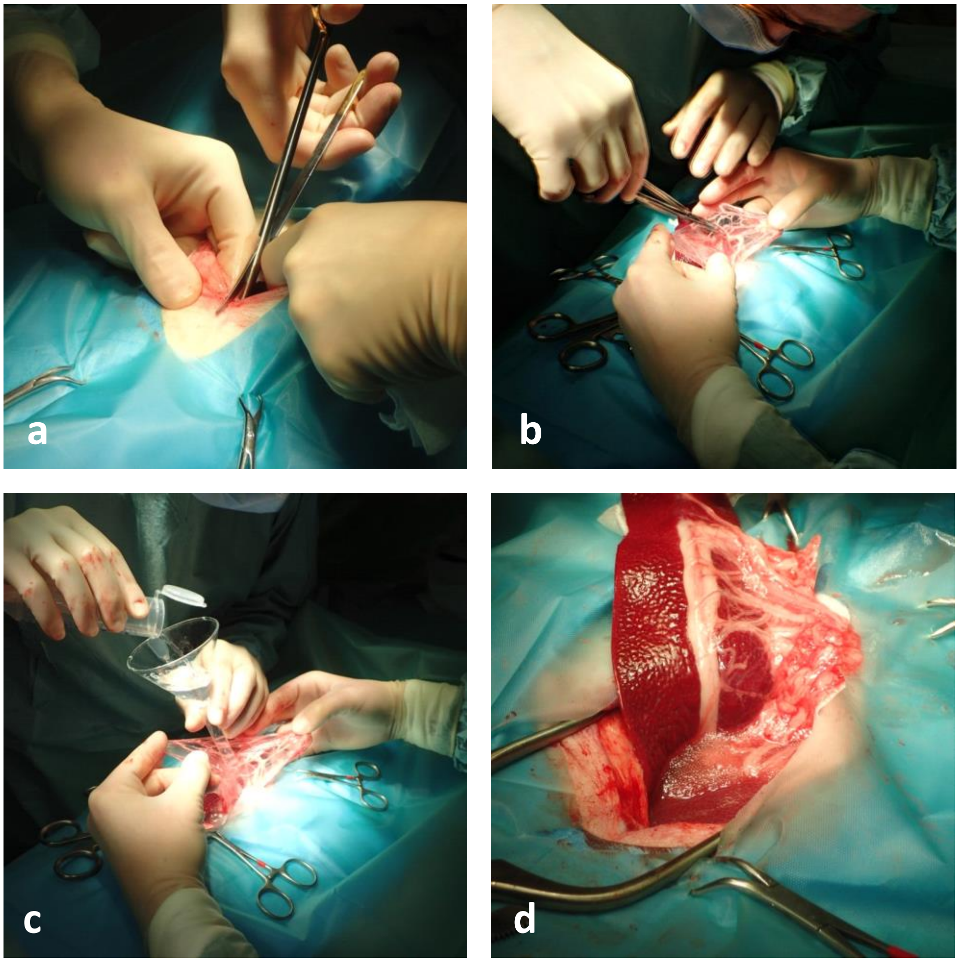

Supplementary Figure 30. Surgical procedures for implantation of microcapsules in pigs. The animal is placed in dorsal recumbency and a sterile midline ventral abdomen site is prepared. (a) An approximately 6 cm midline incision is made immediately cranial to the umbilicus. (b) The spleen and greater omentum are exteriorized and produce an opening in a non-vascular portion of the omentum. (c) The microcapsules are transplanted through this omental opening. (d) The microcapsules will generally stay in the omentum location, closure of the omentum is not necessary. The abdominal tissues are replaced in the abdomen and the midline incision is closed.

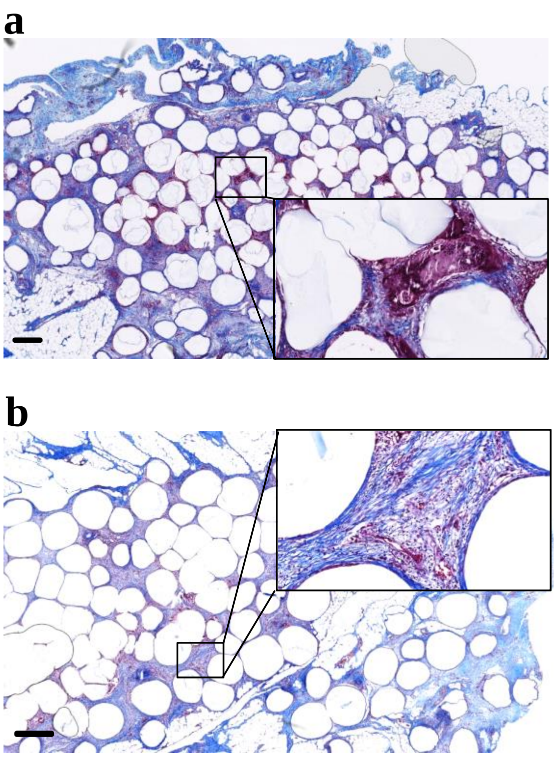

Supplementary Figure 31. Representative Masson's trichrome staining (and a higher magnification) images of retrieved (a) SLG20 and (b) SB-SLG20 alginate microcapsules, 1-month post implantation into the pig omental bursa (Scale bar, 500 μm).

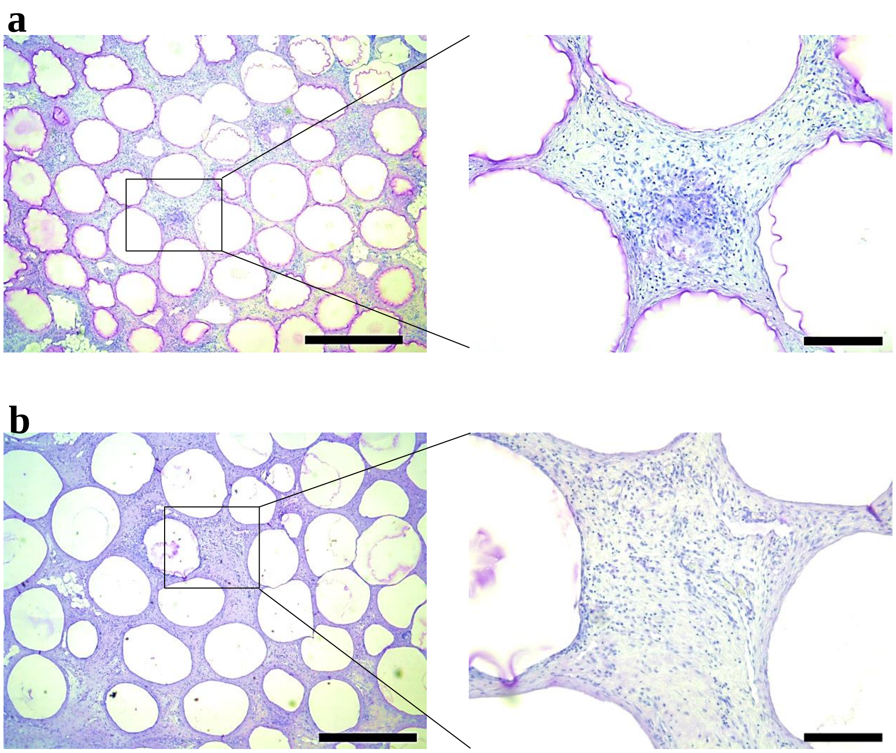

Supplementary Figure 32. PAS-stained histology (and a higher magnification) images of retrieved (a) SLG20 and (b) SB-SLG20 alginate microcapsules, 1-month post implantation into the pig omental bursa (Scale bars, 1 mm on the left and 200 μm on the right).

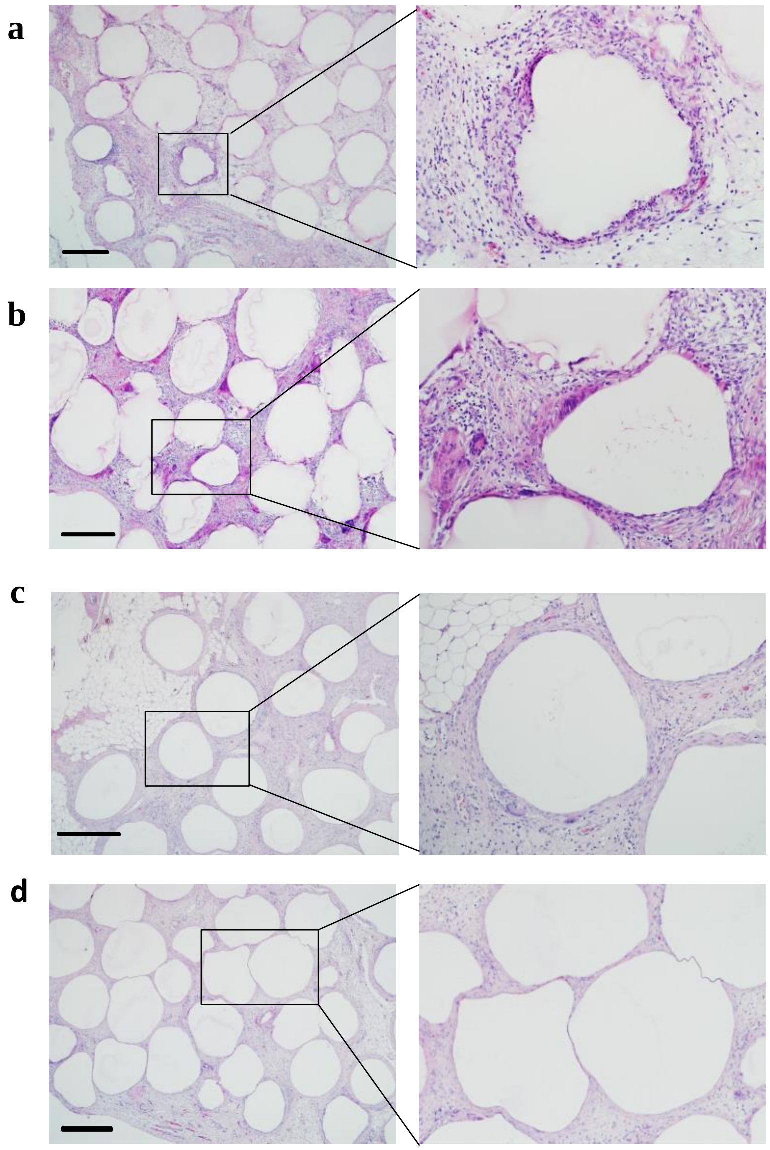

Supplementary Figure 33. Representative H&E stained cross-sectional (and a higher magnification) images of retrieved (a and b; n = 2) SLG20 microcapsules and (c and d; n = 2) SB-SLG20 microcapsules, 1-month post implantation into the pig omental bursa (Scale bar, 500 μm).

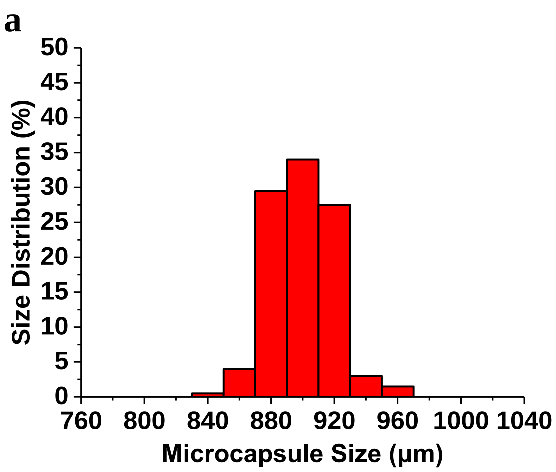

Supplementary Figure 34. Size distribution of the (a) first batch and (b) second batch of islet-containing SB-SLG20 microcapsules before implantation.

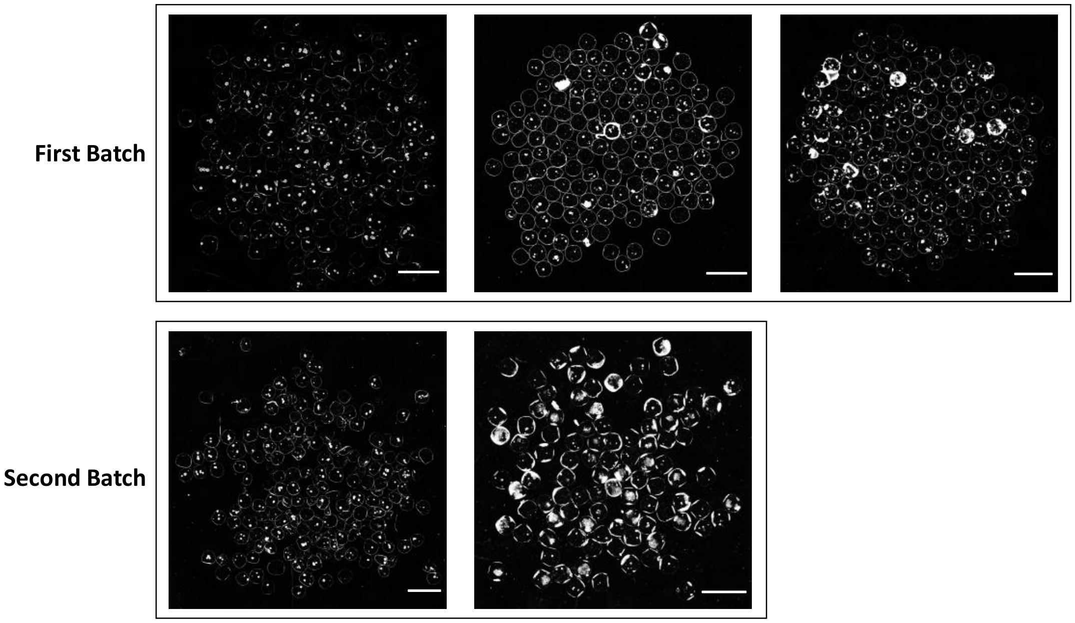

Supplementary Figure 35. Phase contrast images of retrieved islet-containing SB-SLG20 alginate microcapsules, 90 d post intraperitoneal implantation in C57BL/6J mice (Scale bars, 2 mm). Each image represents all microcapsules retrieved from 1 mouse, and each panel represents one batch of experiment.

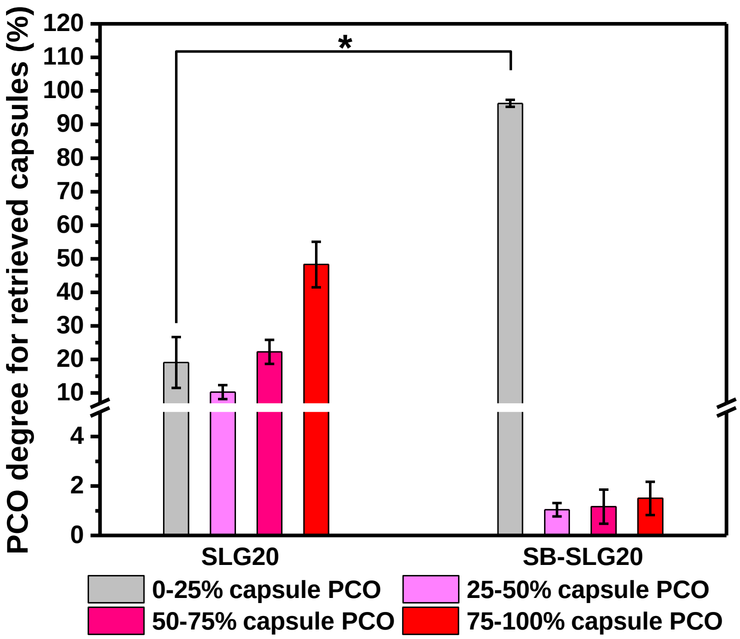

Supplementary Figure 36. Quantification of PCO for retrieved islet-containing microcapsules, 90 d post intraperitoneal implantation in C57BL/6J mice (n = 6 per group). The data are Mean ± SEM, and error bars indicate the SEM. Chi-squared test with Bonferroni correction is used to compare modified alginates to SLG20 alginate. *$P < 0.05$

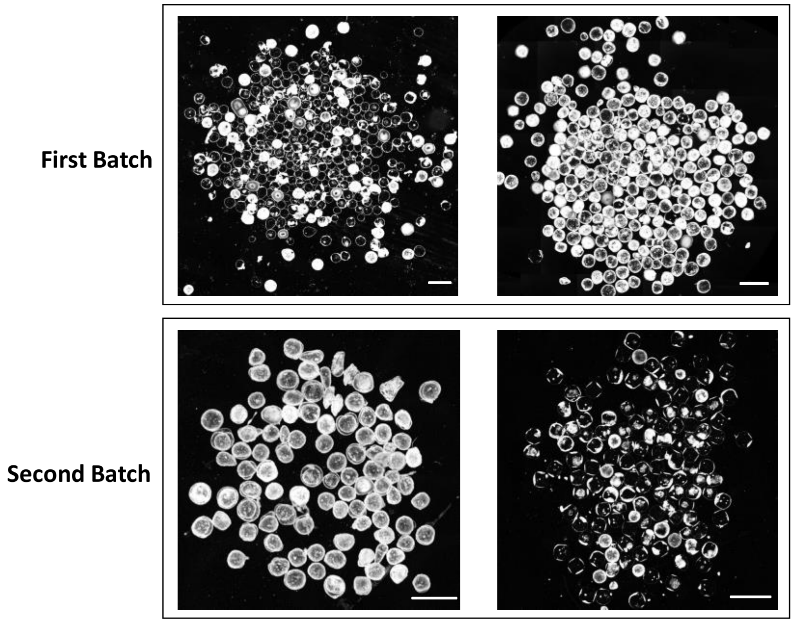

Supplementary Figure 37. Phase contrast images of retrieved islet-containing SLG20 alginate microcapsules, 90 d post intraperitoneal implantation in C57BL/6J mice (Scale bars, 2 mm). Each image represents all microcapsules retrieved from 1 mouse, and each panel represents one batch of experiment.

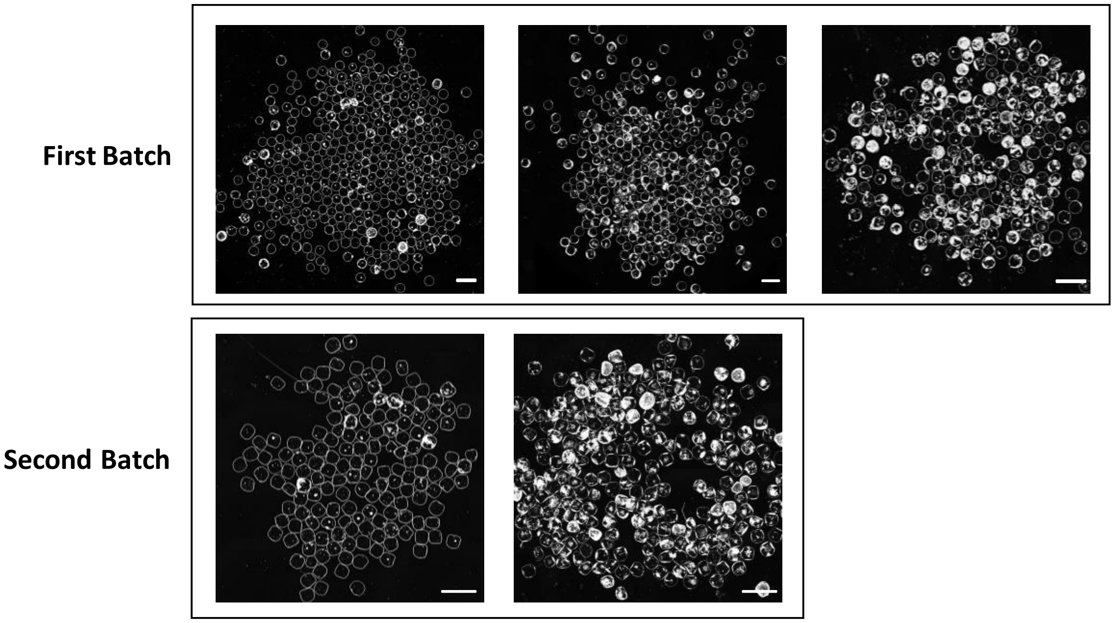

Supplementary Figure 38. Phase contrast images of retrieved islet-containing SB-SLG20 alginate microcapsules, 200 d post intraperitoneal implantation in C57BL/6J mice (Scale bars, 2 mm). Each image represents all microcapsules retrieved from 1 mouse, and each panel represents one batch of experiment.

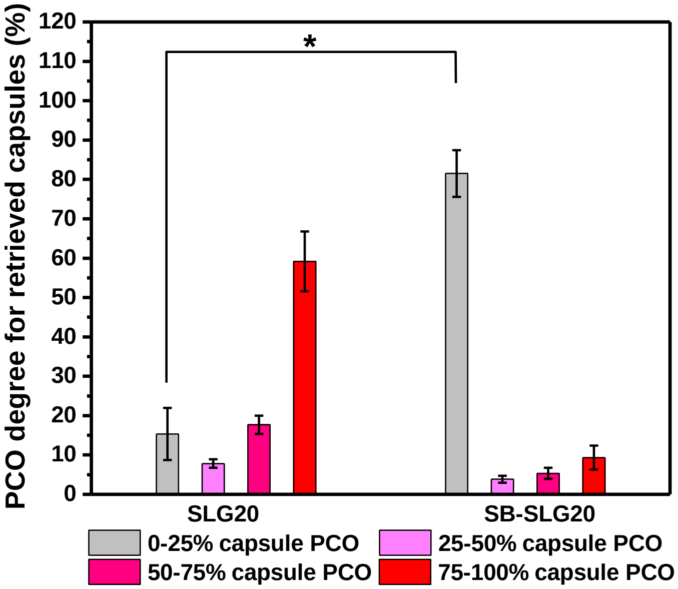

Supplementary Figure 39. Quantification of PCO for retrieved islet-containing microcapsules, 200 d post intraperitoneal implantation in C57BL/6J mice (n = 6 per group). The data are Mean ± SEM, and error bars indicate the SEM. Chi-squared test with Bonferroni correction is used to compare modified alginates to SLG20 alginate. *$P < 0.05$.

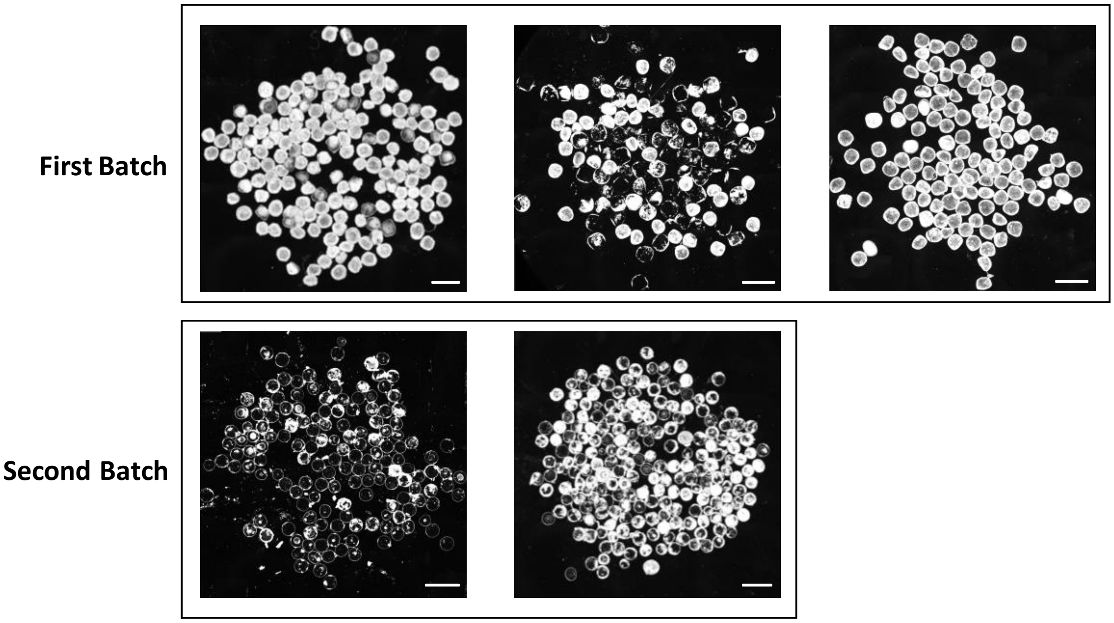

Supplementary Figure 40. Phase contrast images of retrieved islet-containing SLG20 alginate microcapsules, 200 d post intraperitoneal implantation in C57BL/6J mice (Scale bars, 2 mm). Each image represents all microcapsules retrieved from 1 mouse, and each panel represents one batch of experiment.

Supplementary References

1 Miyazaki, S., Ishikawa, F., Fujikawa, T., Nagata, S. & Yamaguchi, K. Intraperitoneal injection of lipopolysaccharide induces dynamic migration of Gr-1high polymorphonuclear neutrophils in the murine abdominal cavity. *Clin. Diagn. Lab. Immunol.* **11**, 452-457 (2004).

2 Zhu, B. *et al.* CD11b+ Ly-6Chi suppressive monocytes in experimental autoimmune encephalomyelitis. *The Journal of Immunology* **179**, 5228-5237 (2007).
