# Supplementary material for: Zwitterionically modified alginates mitigate cellular overgrowth for cell encapsulation
Source: Nat Commun. 2019 Nov 20;10:5262. doi: 10.1038/s41467-019-13238-7 (PMC6868136; doi:10.1038/s41467-019-13238-7)
Supplement: Supplementary file 4 — Reporting Summary [file 41467_2019_13238_MOESM4_ESM.pdf]

nature research

Corresponding author(s): Minglin Ma

Last updated by author(s): Oct 22, 2019

Reporting Summary

Nature Research wishes to improve the reproducibility of the work that we publish. This form provides structure for consistency and transparency in reporting. For further information on Nature Research policies, see Authors & Referees and the Editorial Policy Checklist.

Statistics

For all statistical analyses, confirm that the following items are present in the figure legend, table legend, main text, or Methods section.

n/a	Confirmed	
☐	☒	The exact sample size (*n*) for each experimental group/condition, given as a discrete number and unit of measurement
☐	☒	A statement on whether measurements were taken from distinct samples or whether the same sample was measured repeatedly
☐	☒	The statistical test(s) used AND whether they are one- or two-sided<br>*Only common tests should be described solely by name; describe more complex techniques in the Methods section.*
☒	☐	A description of all covariates tested
☐	☒	A description of any assumptions or corrections, such as tests of normality and adjustment for multiple comparisons
☐	☒	A full description of the statistical parameters including central tendency (e.g. means) or other basic estimates (e.g. regression coefficient) AND variation (e.g. standard deviation) or associated estimates of uncertainty (e.g. confidence intervals)
☐	☒	For null hypothesis testing, the test statistic (e.g. *F*, *t*, *r*) with confidence intervals, effect sizes, degrees of freedom and *P* value noted<br>*Give P values as exact values whenever suitable.*
☒	☐	For Bayesian analysis, information on the choice of priors and Markov chain Monte Carlo settings
☒	☐	For hierarchical and complex designs, identification of the appropriate level for tests and full reporting of outcomes
☒	☐	Estimates of effect sizes (e.g. Cohen's *d*, Pearson's *r*), indicating how they were calculated

Our web collection on statistics for biologists contains articles on many of the points above.

Software and code

Policy information about availability of computer code

Data collection	EVOS AMF4300 imaging system was used to collect phase-contrast images of retrieved alginate microcapsules. Fluorescence Images were captured under a Zeiss LSM710 confocal microscope. Nuclear Magnetic Resonance (NMR) data was collected by Mnova 12 software.
Data analysis	Origin 8 and GraphPad Prism 6 software were used for data plotting and some of the statistical analysis. Mnova12 software was used to analyze the NMR results. IMageJ software was used to analyze protein adsorption results.

For manuscripts utilizing custom algorithms or software that are central to the research but not yet described in published literature, software must be made available to editors/reviewers. We strongly encourage code deposition in a community repository (e.g. GitHub). See the Nature Research guidelines for submitting code & software for further information.

Data

Policy information about availability of data

All manuscripts must include a data availability statement. This statement should provide the following information, where applicable:

- Accession codes, unique identifiers, or web links for publicly available datasets
- A list of figures that have associated raw data
- A description of any restrictions on data availability

The data that support the findings of this study are available from the corresponding author upon request.

Field-specific reporting

Please select the one below that is the best fit for your research. If you are not sure, read the appropriate sections before making your selection.

☒ Life sciences ☐ Behavioural & social sciences ☐ Ecological, evolutionary & environmental sciences

For a reference copy of the document with all sections, see nature.com/documents/nr-reporting-summary-flat.pdf

Life sciences study design

All studies must disclose on these points even when the disclosure is negative.

Sample size	A large number of mice (137 in total) were used in our work to demonstrated the robustness and reproducibility of the cellular overgrowth (CO)-mitigating effect. The sample size for each experimental group was chosen based on previous experience and literature. For large animal studies, we chosen a minimal number (n=2 or 3) of dogs and pigs per experimental group due to resource limitation at the time and ethical concerns.
Data exclusions	No animals were excluded from the analyses.
Replication	We analyzed all microcapsules and presented all replication data in main manuscript and supplemental information to show the robustness of the cellular overgrowth (CO)-mitigating effect.
Randomization	All the samples were randomly assigned to groups.
Blinding	For mouse studies, no blinding was applied. For 1-month dog study and pig study, all the alginate microcapsules were implanted, retrieved and characterized by trained individuals who were blinded to the study. For the 3-month dog study, no blinding was applied.

Reporting for specific materials, systems and methods

We require information from authors about some types of materials, experimental systems and methods used in many studies. Here, indicate whether each material, system or method listed is relevant to your study. If you are not sure if a list item applies to your research, read the appropriate section before selecting a response.

Materials & experimental systems

n/a	Involved in the study	
☐	☒	Antibodies
☒	☐	Eukaryotic cell lines
☒	☐	Palaeontology
☐	☒	Animals and other organisms
☒	☐	Human research participants
☒	☐	Clinical data

Methods

n/a	Involved in the study	
☒	☐	ChIP-seq
☒	☐	Flow cytometry
☒	☐	MRI-based neuroimaging

Antibodies

Antibodies used	Rabbit anti-insulin antibodies (Cat. #ab63820) was purchased from Abcam.<br>Alexa Fluo 594-conjuaged donkey anti-rabbit igG (Cat. #A-21207) was purchased from Invitrogen.<br>α-Smooth Muscle-Cy3 was purchased from Sigma-Aldrich (Cat. # C6198).<br>Anti-mouse CD68-AF488 was purchased from BioLegend (Cat. #137012).<br>Anti-mouse F4/80 was purchased from ThermoFisher (Cat. MF48000).<br>Anti-CD11b (Cat. #ab133357) was purchased from Abcam.<br>Anti-mouse Ly-6G/Ly-6 (Cat. #108419) was purchased from BioLegend.<br>Proteome profiler array kit (Mouse Cytokine Array Panel A; Cat. #ARY006) was purchased from R&D Systems.
Validation	Rabbit anti-insulin antibodies (Cat. #ab63820) gave a positive staining pattern in formalin-fixed paraffin-embedded Mouse Pancreas tissue section and Human pancreas tissue section. Manufacture website: https://www.abcam.com/insulin-antibody-ab63820.html Relevant literature: Kong S et al. Kill two birds with one stone: making multi-transgenic pre-diabetes mouse models through insulin resistance and pancreatic apoptosis pathogenesis. PeerJ 6:e4542 (2018).<br><br>Manufacture website for Alexa Fluo 594-conjuaged donkey anti-rabbit igG (Cat. #A-21207): https://www.thermofisher.com/antibody/product/Donkey-anti-Rabbit-IgG-H-L-Highly-Cross-Adsorbed-Secondary-Antibody-Polyclonal/A-21207<br><br>α-Smooth Muscle-Cy3.<br>It reacts specifically with α-smooth muscle actin in immunoblotting assays and labels smooth muscle cells in frozen or formalin-fixed, paraffin-embedded tissue sections. Manufacture website: https://www.sigmaaldrich.com/catalog/product/sigma/c6198?

lang=en&region=US

Anti-mouse F4/80
The F4/80 antigen is expressed on a wide range of mature tissue macrophages. Manufacture website: https://www.thermofisher.com/antibody/product/F4-80-Antibody-clone-BM8-Monoclonal/MF48000

Anti-CD11b
Manufacture website: https://www.abcam.com/cd11b-antibody-epr1344-ab133357.html

Anti-mouse Ly-6G/Ly-6C
Manufacture website: https://www.biolegend.com/en-us/products/alexa-fluor-488-anti-mouse-ly-6g-ly-6c-gr-1-antibody-2711

Anti-mouse CD68-AF488
Manufacture website: https://www.biolegend.com/en-us/products/alexa-fluor-488-anti-mouse-cd68-antibody-6619

Proteome profiler array kit (Mouse Cytokine Array Panel A)
Manufacture website: https://www.rndsystems.com/cn/products/proteome-profiler-mouse-cytokine-array-kit-panel-a_ary006

Animals and other organisms

Policy information about studies involving animals; ARRIVE guidelines recommended for reporting animal research

Laboratory animals	Sprague-Dawley rats (male, strain code 400, 250-275 g weight, and from Charles River).<br>C57BL/6J mice (male, stain code: 00664, 8-12 weeks, and from Jackson Laboratory)<br>Beagle dogs (female, 10 months to 1.5 years in age, and from Marshall Bioresources)<br>Göttingen minipigs (female, 8 months in age)
Wild animals	The study did not involve wild animals.
Field-collected samples	The study did not involve samples collected from the field.
Ethics oversight	All animal procedures using C57BL/6J mice, Sprague Dawley rats, and Beagle dogs were approved by and performed according to the guidelines of the Institutional Animal Care and Use Committee at the Cornell University.<br>Göttingen minipigs experiment was performed at Novo Nordisk A/S, and the protocols were approved by the Danish Animal Experimentation Inspectorate and carried out by trained and licensed personnel.

Note that full information on the approval of the study protocol must also be provided in the manuscript.
